# Global burden of low back pain and its attributable risk factors from 1990 to 2021: a comprehensive analysis from the global burden of disease study 2021

**DOI:** 10.3389/fpubh.2024.1480779

**Published:** 2024-11-13

**Authors:** Yue Li, Congying Zou, Weidong Guo, Feng Han, Tao Fan, Lei Zang, Guoshun Huang

**Affiliations:** ^1^Department of Orthopedic Surgery, General Hospital of Tisco, Taiyuan, Shanxi, China; ^2^Department of Orthopedic Surgery, Beijing Chao-yang Hospital, Capital Medical University, Beijing, China

**Keywords:** low back pain, global burden of disease, age-standardized incidence rate, age-standardized disability-adjusted life years rate, risk factors

## Abstract

**Background:**

This study aimed to examine the evolving trends in the global burden of low back pain (LBP) from 1990 to 2021 and predicted disease burden until 2035.

**Materials and methods:**

LBP-related data were obtained from the Global Health Data Exchange (GHDx) query tool. All estimates and their 95% uncertainty intervals (UIs) were generated using DisMod-MR 2.1, a Bayesian meta-regression tool in Global Burden of Disease, Injury, and Risk Factor Study (GBD) 2021. Data processing and visualization were conducted using The R Programming Language software version 4.3.2 and Joinpoint 4.7.

**Results:**

In 2021, approximately 628.8 million people worldwide were affected by LBP, with approximately 266.9 million new incident cases and age-standardized incidence rate (ASIR) of 3176.6 per 100,000. Compared with 1990, although the ASIR and age-standardized disability-adjusted life years rate (ASDALYsR) decreased, absolute numbers increased significantly. Projections for 2035 reveal a continued decline in ASIR and ASDALYsR for LBP. The LBP burden varied by the sociodemographic index quintile and GBD region, with the highest ASIR and ASDALYsR observed in Central Europe and the greatest decrease in East Asia. Globally, women bear a higher burden of LBP than men, with middle-aged populations experiencing the heaviest burden. Occupational ergonomic factors, high body mass index, and smoking remain the primary risk factors for LBP, with occupational ergonomic factors contributing the most to the overall burden.

**Conclusion:**

Despite a projected decline in incidence, the global burden of LBP persists, exhibiting significant regional and gender disparities. To mitigate its future burden, precise and effective prevention and control strategies targeting high-risk factors are imperative.

## Introduction

Low back pain (LBP) is characterized as discomfort in the back, extending from the lower margin of the 12th rib (Thoracic rib) to the subgluteal fold, and may be accompanied by pain in one or both lower extremities, persisting for a minimum duration of 1 day (1). Acute episodes of LBP typically resolve spontaneously, while chronic LBP is considered to result from a multifaceted interplay of biological, psychological, and social determinants (2). The Global Burden of Disease, Injury, and Risk Factor Study (GBD) systematically quantifies health loss attributable to a multitude of diseases and injuries across ages, sexes, years, and geographic regions (1). The GBD 2019 report indicated a substantial global increase in the number of prevalent cases, incident cases, and years lived with disability (YLDs) due to LBP from 1990 to 2019, and LBP remained the foremost cause of YLDs worldwide in 2019 (3).

The current burden and attributable risk factors for LBP are essential for devising effective control and prevention strategies. The global burden of LBP has been documented in three studies using GBD study data in 2010, 2017, and 2019 (3–5). GBD 2021 (6) showed that LBP was the ninth leading cause of DALYs, and the burden of disease remains high worldwide. However, no recent study has comprehensively detailed multiple factors such as incidence, disability-adjusted life years (DALYs), and major risk factors of LBP across regions. Therefore, this study, based on the most recent estimates from the GBD 2021, aimed to extend and update previous research to provide comparable and comprehensive information on the global burden of LBP and its attributable risk factors from 1990 to 2021 and predict future trends in LBP development.

## Materials and methods

### Data sources

Raw data of LBP were collected by sex, region, and country from 1990 to 2021 using the Global Health Data Exchange (GHDx) query tool (6).^1^ In the GBD 2021 study, estimates of disease and injury incidence and burden were derived using DisMod-MR 2.1, a Bayesian meta-regression tool developed by the GBD research group.

### Data definition and collection

Data and uncertainty intervals (UI) for new incident cases, prevalent cases, and DALYs associated with LBP in different countries, regions, ages, and sexes were accessed and collected from the GBD 2021 databases. LBP related rates were expressed using the age-standardized rate (ASR), which was also obtained directly from the database, including the age-standardized incidence rate (ASIR), age-standardized DALYs rate (ASDALYsR). YLDs quantify nonfatal health losses, Years of life lost due to premature mortality (YLLs) quantify fatal health losses, and DALYs represent the sum of years lost because of premature death and YLDs. DALYs equals the sum of YLLs and YLDs; because LBP does not result in mortality, the DALYs estimate in this study is equivalent to YLDs (4).

The Sociodemographic Index (SDI), a composite measure of developmental status strongly associated with health outcomes, was developed by GBD researchers, and used to help generate these estimates. According to the SDI, countries and regions were divided into five: low, low–medium, medium, high–medium, and high. The world was also divided into 21 GBD regions according to geographical factors.

### Statistical analysis

We used The R Programming Language (R) (version 4.3.2) and Joinpoint software (version 4.7) to perform data processing and visualization. Estimated annual percentage change (EAPC) is a widely accepted measure for summarizing ASR trends over specific intervals. The regression line was fitted to the natural logarithm of the ASR ratio, i.e., y = *α* + *β*x + ɛ, where y = ln (ASR) and x = calendar year (7). EAPC was calculated as 100 × (exp (β) -1), with its 95% confidence interval (CI) derived from a linear regression model (8). The ASRs were considered increasing if both the EAPC estimate and the lower limit of its 95% CI were > 0. Conversely, ASRs were deemed to be decreasing if the upper limit of both the EAPC estimate and its 95% CI were < 0. Otherwise, ASRs were considered stable over time.

Occupational ergonomic factors, high BMI, and smoking were risk factors included in GBD 2021, and relevant factors were defined and measured from a previous study (1). In this study, analysis was performed using R version 4.3.2 after obtaining data from the database.

Trend analysis of ASIR and ASDALYsR was conducted using Joinpoint 4.7, developed by the National Cancer Institute.^2^ The Joinpoint regression model is a statistical analysis method widely used to examine trends in disease incidence or mortality over time (9–11). The core principle of Joinpoint regression is to analyze whether APC and AAPC values are significant by identifying the inflection points, or join points, in the model. These inflection points segment the long-term trend of ASIR and ASDALYsR into several intervals, each described by APC and AAPC, thereby revealing the change patterns of ASIR and ASDALYsR over time. *p* < 0.05 was considered significantly different in this study.

Predicted images were drawn by the following method. Standard-age population data (12) and predicted population data for 2100 (13) were obtained from the relevant literature. Standard-age population data, predicted population data for 2,100, incident cases for each age group from 1990 to 2021, and DALYs of each age group from 1990 to 2021 were compiled using R version 4.3.2 and projected with the age spec, the rate function of the BAPC package. The BAPC package employs the integrated nested Laplacian approximation for complete Bayesian inference. Image rendering was performed using the plot BAPC function within the BAPC package of R.

### Results

In 2021, a total of 628.8 million (95% UI, 551.8–700.9) people worldwide were affected by LBP (Supplementary Table S1), whereas the incident cases for LBP globally were approximately 266.9 million (95% UI, 235.4–299.4) with an ASIR of [3176.6 (95% UI, 2811.8–3562.3) per 100,000 people] (Table 1). In 1990, 386.7 million (95% UI, 341.6–434.2) people worldwide were affected by LBP (Supplementary Table S1), and the global incident cases of LBP were 165.1 million (95% UI, 145.8–185.9) with an ASIR of [3535.0 (95% UI, 3133.0–3961.0) per 100,000 people]. The EAPC in ASIR for LBP was −0.29% (95% CI, −0.32 to −0.26) from 1990 to 2021 (Table 1).

**Table 1:** Incidence of low back pain in 1990 and 2021 for both sexes in regions, with EAPC from 1990 to 2021.

Location	1990 counts	1990 ASR per 100,000 people	2021 counts	2021 ASR per 100,000 people	EAPC in ASR 1990–2021 (95% CI)
Sex					
Male	63,553,575 (55,765,751 to 71,904,401)	2770.8 (2441.21 to 3122.36)	100,775,918 (88,485,948 to 113,863,884)	2450.55 (2161.03 to 2758.87)	−0.35% (−0.38 to −0.33)
Female	101,510,307 (90,057,184 to 114,008,585)	4263.66 (3774.25 to 4776.07)	166,097,403 (146,916,668 to 186,192,888)	3879.94 (3438.63 to 4344.9)	−0.24% (−0.28 to −0.2)
Global	165,063,882 (145,785,269 to 185,933,884)	3534.99 (3133.04 to 3960.99)	266,873,321 (235,369,489 to 299,406,380)	3176.63 (2811.82 to 3562.29)	−0.29% (−0.32 to −0.26)
SDI quintiles					
High SDI	43,722,165 (39,062,418 to 49,065,394)	4424.22 (3944.67 to 4976.02)	59,095,839 (52,994,576 to 65,248,529)	4118.8 (3695.02 to 4589.31)	−0.17% (−0.19 to −0.14)
High-middle SDI	39,553,208 (35,006,678 to 44,407,273)	3734.53 (3318.15 to 4178.19)	54,050,238 (47,424,129 to 60,795,832)	3250.38 (2870.7 to 3648.88)	−0.38% (−0.42 to −0.33)
Middle SDI	42,247,526 (37,048,076 to 47,853,836)	3016.06 (2660.6 to 3392.23)	74,568,788 (65,108,174 to 84,107,629)	2770.57 (2435.96 to 3119.83)	−0.19% (−0.24 to −0.15)
Low-middle SDI	28,310,723 (24,878,028 to 32,058,294)	3289.8 (2902.59 to 3692.6)	54,298,819 (47,620,836 to 61,354,906)	3113.93 (2740.21 to 3499.77)	−0.18% (−0.22 to −0.13)
Low SDI	11,039,527 (9,652,870 to 12,437,704)	3315.99 (2931.45 to 3721.91)	24,608,330 (21,598,995 to 27,916,670)	3134.89 (2760.73 to 3519.33)	−0.19% (−0.21 to −0.17)
GBD regions					
Andean Latin America	736,141 (647,842 to 830,131)	2520.72 (2220.84 to 2846.49)	1,646,434 (1,458,880 to 1,844,797)	2537.33 (2248.54 to 2847.88)	0.04% (0.01 to 0.06)
Australasia	1,116,813 (995,033 to 1,264,046)	5066.6 (4532.9 to 5758.99)	1,793,375 (1,584,866 to 1,997,436)	4715.64 (4181.41 to 5288.91)	−0.16% (−0.18 to −0.13)
Caribbean	823,557 (721,753 to 927,447)	2649.01 (2327.25 to 2988.32)	1,338,623 (1,184,999 to 1,505,225)	2618.94 (2316.54 to 2943.07)	−0.02% (−0.03 to −0.01)
Central Asia	2,219,225 (1,967,968 to 2,496,734)	3946.39 (3493.46 to 4422.79)	3,627,338 (3,190,645 to 4,122,873)	3908.43 (3443.95 to 4408.07)	−0.03% (−0.03 to −0.02)
Central Europe	7,329,411 (6,501,413 to 8,246,292)	5279.12 (4690.7 to 5941.73)	8,079,891 (7,097,942 to 9,076,612)	5181.24 (4595.33 to 5834.52)	−0.07% (−0.08 to −0.07)
Central Latin America	4,022,190 (3,510,176 to 4,572,563)	3140.4 (2773.02 to 3538.98)	8,328,520 (7,336,429 to 9,409,867)	3173.31 (2801.57 to 3585.76)	0.03% (−0.01 to 0.07)
Central Sub-Saharan Africa	1,181,032 (1,029,213 to 1,339,383)	3359.32 (2938.81 to 3787.45)	2,999,484 (2,638,593 to 3,421,283)	3259.03 (2879.66 to 3674.29)	−0.12% (−0.14 to −0.09)
East Asia	31,011,647 (27,123,453 to 35,291,824)	2866.16 (2520.47 to 3229.74)	45,417,237 (39,382,941 to 51,288,188)	2369.25 (2088.98 to 2663.65)	−0.44% (−0.53 to −0.35)
Eastern Europe	12,246,259 (10,800,139 to 13,742,205)	4752.91 (4216.92 to 5309.17)	12,839,596 (11,238,456 to 14,387,501)	4619.12 (4112.22 to 5173.33)	−0.04% (−0.06 to −0.03)
Eastern Sub-Saharan Africa	3,920,767 (3,442,324 to 4,415,532)	3349.44 (2952.44 to 3770.07)	9,141,988 (8,019,263 to 10,354,866)	3238.98 (2855.2 to 3641.36)	−0.12% (−0.13 to −0.11)
High-income Asia Pacific	9,006,130 (7,938,409 to 10,123,637)	4629.55 (4106.23 to 5216.54)	11,053,079 (9,710,908 to 12,353,921)	4236.84 (3745.6 to 4769.29)	−0.23% (−0.25 to −0.21)
High-income North America	14,670,611 (13,042,429 to 16,545,457)	4712.08 (4197.89 to 5302.41)	20,525,968 (18,564,440 to 22,433,456)	4430.69 (4019.85 to 4856.34)	−0.07% (−0.12 to −0.01)
North Africa and Middle East	9,687,730 (8,533,318 to 10,892,164)	3762.42 (3358.17 to 4228.9)	21,554,211 (18,949,089 to 24,589,228)	3686.08 (3259.35 to 4157.55)	−0.05% (−0.06 to −0.04)
Oceania	126,038 (109,329 to 143,469)	2756.62 (2423.35 to 3122.59)	297,549 (259,577 to 339,195)	2728.63 (2394.39 to 3098.38)	−0.02% (−0.03 to 0)
South Asia	26,843,747 (23,552,246 to 30,395,436)	3285.69 (2895.16 to 3689.97)	51,725,612 (45,244,519 to 58,532,118)	2970.33 (2606.51 to 3334.99)	−0.33% (−0.42 to −0.24)
Southeast Asia	9,092,801 (7,980,280 to 10,181,926)	2563.77 (2263.65 to 2880.72)	18,102,798 (15,777,830 to 20,544,107)	2518.34 (2218.52 to 2844.11)	−0.04% (−0.05 to −0.03)
Southern Latin America	2,000,972 (1,776,610 to 2,262,609)	4169.07 (3705.78 to 4713.46)	3,175,959 (2,802,991 to 3,549,723)	4138.35 (3660.72 to 4648.24)	−0.01% (−0.05 to 0.03)
Southern Sub-Saharan Africa	1,085,155 (954,299 to 1,220,274)	2946.99 (2592.52 to 3308.26)	2,000,245 (1,750,711 to 2,271,266)	2810.63 (2465.1 to 3178.51)	−0.13% (−0.14 to −0.12)
Tropical Latin America	4,863,631 (4,256,075 to 5,541,750)	3780.7 (3331.84 to 4274.24)	9,654,115 (8,506,642 to 10,873,405)	3857.44 (3402.33 to 4336.37)	0.04% (0.02 to 0.06)
Western Europe	19,199,804 (17,127,530 to 21,392,148)	4173.12 (3732.89 to 4684.76)	23,987,309 (21,198,828 to 26,893,840)	4040.66 (3579.55 to 4541.58)	−0.06% (−0.07 to −0.04)
Western Sub-Saharan Africa	3,880,221 (3,401,179 to 4,375,940)	3054.43 (2693.49 to 3440.05)	9,583,990 (8,386,342 to 10,870,298)	2974.28 (2616.26 to 3351.94)	−0.1% (−0.14 to −0.06)

Data in parentheses are 95% uncertainty interval unless specifically noted. ASR, Age Standardized Incidence Rate; EAPC, Estimated Annual Percentage Change; CI, Confidence interval; SDI, Sociodemographic Index.

Across SDI quintiles, in 2021, the highest ASIR and ASDALYsR of LBP was seen in the high SDI quintile [4118.80 (95% UI, 3695.02–4589.31) per 100,000 people] and [1094.34 (95% UI, 792.26–1459.42) per 100,000 people]. The largest decrease in ASIR was in the high-middle SDI quintile with an EAPC of −0.38% (95% CI, −0.42 to −0.33). In GBD regions, the largest decrease in ASIR was in East Asia with an EAPC of −0.44 (95% CI, −0.53 to −0.35) (Table 1).

Globally, the LBP-related DALYs in 2021 was 70.2 million (95% UI, 50.2–94.1) with an ASDALYsR of [832.2 (95% UI, 595.9–1115.2) per 100,000 people]. In 1990, it was 43.4 million (95% UI, 31.1–58.4) with an ASDALYsR of [937.3 (95% UI, 669.1–1261.0) per 100,000 people]. From 1990 to 2021 LBP had a decreased trend in ASDALYsR with an EAPC of −0.32% (95% CI, −0.35 to −0.28). In SDI quintile, the largest decrease in ASDALYsR was in the high-middle SDI quintile with an EAPC of −0.41% (95% CI, −0.46 to −0.37). In GBD regions, the largest decrease in ASDALYsR was in East Asia with an EAPC of −0.46 (95% CI, −0.56 to −0.36) (Table 2).

**Table 2:** DALYs of low back pain in 1990 and 2021 for both sexes in regions, with EAPC from 1990 to 2021.

Location	1990 counts	1990 ASR per 100,000 people	2021counts	2021 ASR per 100,000 people	EAPC in ASR 1990–2021 (95% CI)
Sex					
Male	16,412,085 (11,713,623 to 22,118,911)	720.6 (512.4 to 974.5)	26,222,007 (18,702,285 to 35,396,348)	635.48 (453.91 to 854.29)	−0.35% (−0.38 to −0.33)
Female	26,974,140 (19,377,113 to 36,203,736)	1142.28 (817.03 to 1533.18)	43,934,955 (31,447,685 to 58,945,143)	1021.52 (732.39 to 1370.45)	0.28% (−0.32 to −0.24)
Global	43,386,226 (31,083,937 to 58,355,210)	937.34 (669.13 to 1,261)	70,156,962 (50,194,205 to 94,104,688)	832.18 (595.85 to 1115.24)	−0.32% (−0.35 to −0.28)
SDI quintiles					
High SDI	11,851,271 (8,465,650 to 15,920,115)	1189.91 (854.78 to 1600.27)	15,937,890 (11,551,525 to 21,216,646)	1094.34 (792.26 to 1459.42)	−0.2% (−0.23 to −0.17)
High-middle SDI	10,524,244 (7,567,872 to 14,235,162)	995.35 (712.29 to 1343.18)	14,394,112 (10,223,002 to 19,435,230)	854.81 (611.67 to 1,147)	−0.41% (−0.46 to −0.37)
Middle SDI	10,911,753 (7,780,236 to 14,604,346)	788.71 (560.36 to 1061.61)	19,437,989 (13,849,605 to 26,187,075)	717.45 (512.59 to 962.53)	−0.2% (−0.25 to −0.15)
Low-middle SDI	7,245,518 (5,146,960 to 9,671,477)	857.76 (613.87 to 1152.2)	14,064,118 (10,073,508 to 18,918,257)	810.23 (580.5 to 1088.22)	−0.17% (−0.22 to −0.12)
Low SDI	2,801,498 (1,997,111 to 3,731,852)	863.78 (617.16 to 1157.85)	6,254,664 (4,446,006 to 8,373,290)	815.25 (582.63 to 1097.15)	−0.18% (−0.2 to −0.16)
GBD regions					
Andean Latin America	185,029 (130,897 to 246,432)	646.13 (458.75 to 865.08)	419,827 (298,545 to 559,565)	646.88 (458.19 to 863.75)	0.03% (0.01 to 0.06)
Australasia	304,861 (215,769 to 408,899)	1376.56 (976 to 1844.16)	488,387 (349,002 to 661,173)	1268.22 (904.77 to 1709.01)	−0.19% (−0.22 to −0.17)
Caribbean	210,760 (151,733 to 281,762)	684.15 (489.1 to 922.21)	344,542 (247,890 to 462,391)	670.86 (483.43 to 901.16)	−0.02% (−0.03 to −0.01)
Central Asia	576,974 (415,109 to 767,337)	1043.07 (747.31 to 1393.92)	952,839 (682,011 to 1,286,067)	1029.87 (732.88 to 1385.72)	−0.02% (−0.03 to −0.02)
Central Europe	2,060,141 (1,476,137 to 2,773,949)	1475.91 (1058.6 to 1976.09)	2,290,522 (1,634,308 to 3,088,294)	1439.39 (1027.23 to 1934.4)	−0.08% (−0.09 to −0.08)
Central Latin America	1,038,273 (731,753 to 1,387,394)	827.1 (589.11 to 1110.66)	2,204,585 (1,574,612 to 2,963,661)	837.42 (597.9 to 1127.03)	0.04% (0 to 0.09)
Central Sub-Saharan Africa	295,144 (209,057 to 395,657)	863.94 (623.33 to 1164.32)	754,976 (536,134 to 1,006,808)	842.65 (603.12 to 1136.37)	−0.09% (−0.12 to −0.07)
East Asia	8,080,257 (5,742,297 to 10,947,390)	751.03 (532.46 to 1014.91)	11,867,556 (8,333,735 to 16,052,098)	611.77 (433.79 to 820.52)	−0.46% (−0.56 to −0.36)
Eastern Europe	3,353,744 (2,399,226 to 4,527,209)	1293.39 (925.73 to 1741.73)	3,508,059 (2,503,644 to 4,720,036)	1241.42 (889.66 to 1666.37)	−0.05% (−0.08 to −0.03)
Eastern Sub-Saharan Africa	987,109 (701,131 to 1,317,166)	872.15 (624.42 to 1168.26)	2,317,834 (1,643,306 to 3,112,633)	844.44 (602.74 to 1135.48)	−0.1% (−0.11 to −0.1)
High-income Asia Pacific	2,466,629 (1,764,723 to 3,339,335)	1256.21 (903.17 to 1688.97)	3,027,728 (2,147,702 to 4,085,674)	1140.12 (814.58 to 1534.15)	−0.27% (−0.29 to −0.24)
High-income North America	3,943,904 (2,820,966 to 5,255,899)	1260.42 (905.37 to 1682.35)	5,425,099 (3,944,504 to 7,058,790)	1159.5 (843.77 to 1513.97)	−0.11% (−0.17 to −0.05)
North Africa and Middle East	2,513,043 (1,774,596 to 3,348,746)	997.39 (719.01 to 1341.15)	5,661,667 (4,030,973 to 7,583,353)	967.37 (696.7 to 1296.96)	−0.07% (−0.09 to −0.05)
Oceania	31,920 (22,753 to 42,740)	712.13 (507.26 to 954.19)	76,195 (54,394 to 102,628)	705.15 (504.09 to 943.46)	−0.01% (−0.02 to 0.01)
South Asia	6,828,442 (4,869,769 to 9,096,399)	849.6 (608.76 to 1141.44)	13,247,628 (9,487,458 to 17,842,552)	762.34 (544.52 to 1022.84)	−0.34% (−0.43 to −0.24)
Southeast Asia	2,326,494 (1,659,013 to 3,122,018)	665.22 (474.6 to 892.67)	4,757,936 (3,371,946 to 6,410,456)	657.99 (467.99 to 885.65)	0% (−0.01 to 0.01)
Southern Latin America	524,866 (373,418 to 706,452)	1097.59 (782.23 to 1475.55)	843,277 (596,115 to 1,134,514)	1090.83 (772.11 to 1464.42)	−0.02% (−0.06 to 0.02)
Southern Sub-Saharan Africa	273,764 (196,708 to 366,348)	758.59 (541.31 to 1017.04)	505,036 (361,967 to 678,388)	712.89 (510.7 to 955.02)	−0.16% (−0.17 to −0.15)
Tropical Latin America	1,264,922 (900,250 to 1,690,841)	998.4 (712.23 to 1339.07)	2,598,774 (1,862,649 to 3,499,223)	1029.57 (741.54 to 1382.07)	0.07% (0.05 to 0.1)
Western Europe	5,145,785 (3,678,724 to 6,891,381)	1105.46 (797.53 to 1482.55)	6,444,977 (4,566,679 to 8,666,045)	1068.98 (766.39 to 1441.34)	−0.07% (−0.08 to −0.05)
Western Sub-Saharan Africa	974,166 (694,695 to 1,306,372)	786.45 (559.82 to 1055.9)	2,419,519 (1,712,962 to 3,244,286)	770.61 (549.43 to 1039.03)	−0.07% (−0.1 to −0.03)

Data in parentheses are 95% uncertainty interval unless specifically noted. ASR, Age Standardized Rate; DALYs, Disability Adjusted Life Years; EAPC, Estimated Annual Percentage Change; CI, Confidence interval; SDI, Sociodemographic Index.

**Figure 1:**
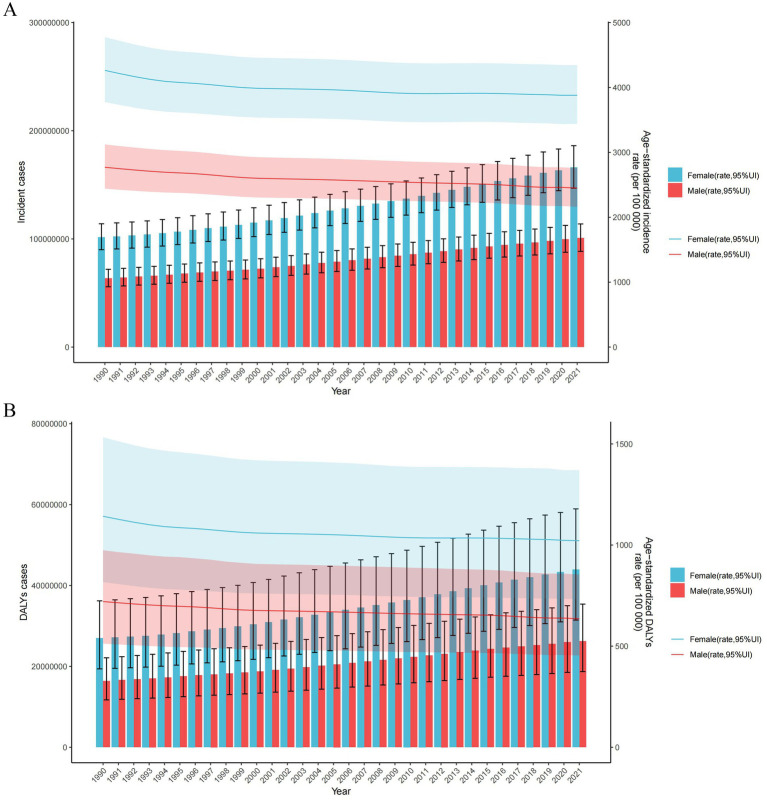
Trends in the number and age-standardized rates of new incident cases (A) and DALYs (B) of LBP worldwide from 1990 to 2021. Error bars represent 95% uncertainty intervals (UIs) of the numbers. Shading indicates the 95% UI of the rate. DALYs, disability-adjusted life years.

By sex, in 2021, more incident cases of LBP were recorded in women than in men, with women also exhibiting a higher ASIR of LBP than men (Figure 1A). In addition, the number of DALYs due to LBP was greater in women than in men (Figure 1B). The absolute EAPC values for ASIR and ASDALYsR of LBP were higher in men than in women in 2021 (Tables 1, 2).

**Figure 2:**
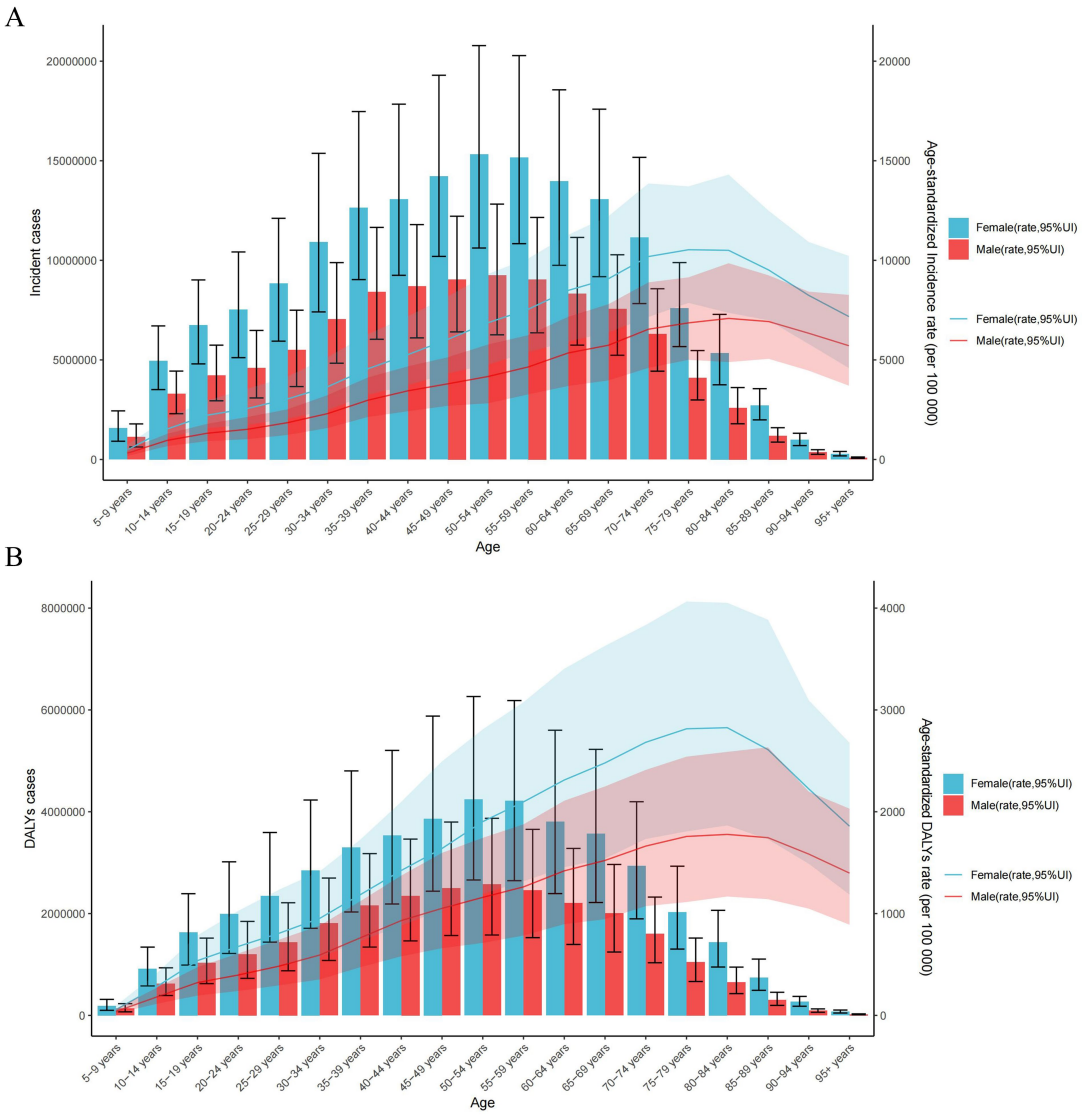
Number and rates of new incident cases of LBP (A) and DALYs cases (B) in different age intervals by sex in 2021. The error bars represent the 95% uncertainty intervals (UIs) of the numbers. Shading indicates the 95% UI of the rate. DALYs, disability-adjusted life years.

Age-specific counts, rates of incident cases, and DALY cases for LBP by sex in 2021 are shown in Figure 2. The incident cases of LBP peaked between the age of 50–54 years in both sexes, and incident cases at any age were higher in women than in men. The number of LBP-related DALYs peaked at the age of 50–54 years in both sexes, and the number of DALYs at any age was higher in women than in men (Figure 2B). The age-specific incident and DALYs rates of LBP increased with age in both sexes until the age of 80–84 years (Figure 2).

**Figure 3:**
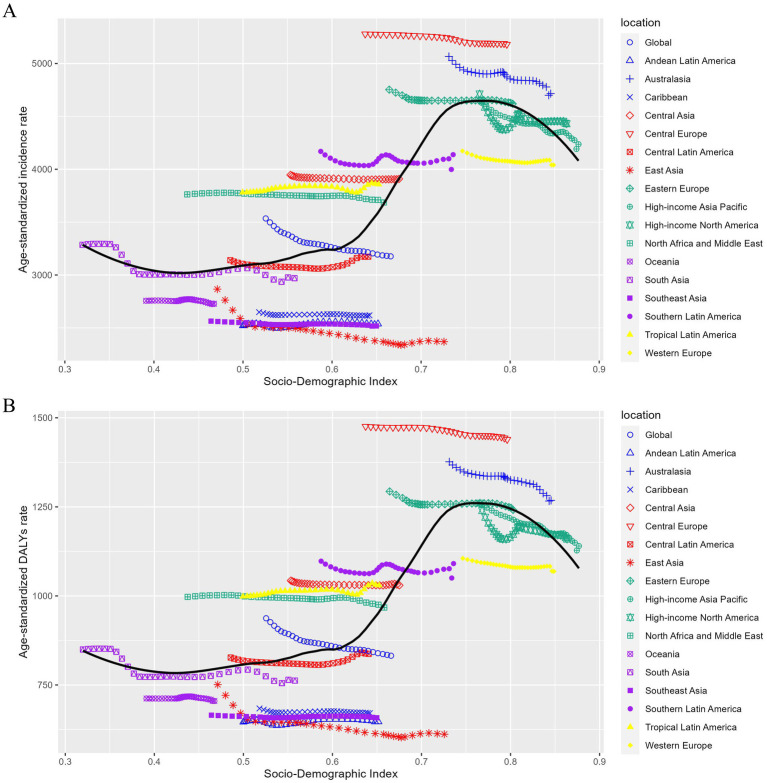
Trends in age-standardized incidence (A) and DALY (B) rates of LBP by sociodemographic index, worldwide, and 21 GBD regions, 1990–2021. For each region, points from the left to right describe estimates for each year from 1990 to 2021. DALYs, disability-adjusted life years.

Across 21 GBD regions, in 2021, the highest ASIR of LBP was seen in Central Europe [5181.24 (95% UI, 4595.33–5834.52) per 100,000 people], whereas the lowest ASIR was observed in East Asia [2369.25 (95% UI, 2088.98–2663.65) per 100,000 people] (Table 1; Figure 3A). The highest ASDALYsR [1439.39 (95% UI, 1027.23–1934.4) per 100,000 people] was observed in Central Europe, whereas the lowest [611.77 (95% UI, 433.79–820.52) per 100,000 people] was seen in East Asia (Table 2; Figure 3B).

**Figure 4:**
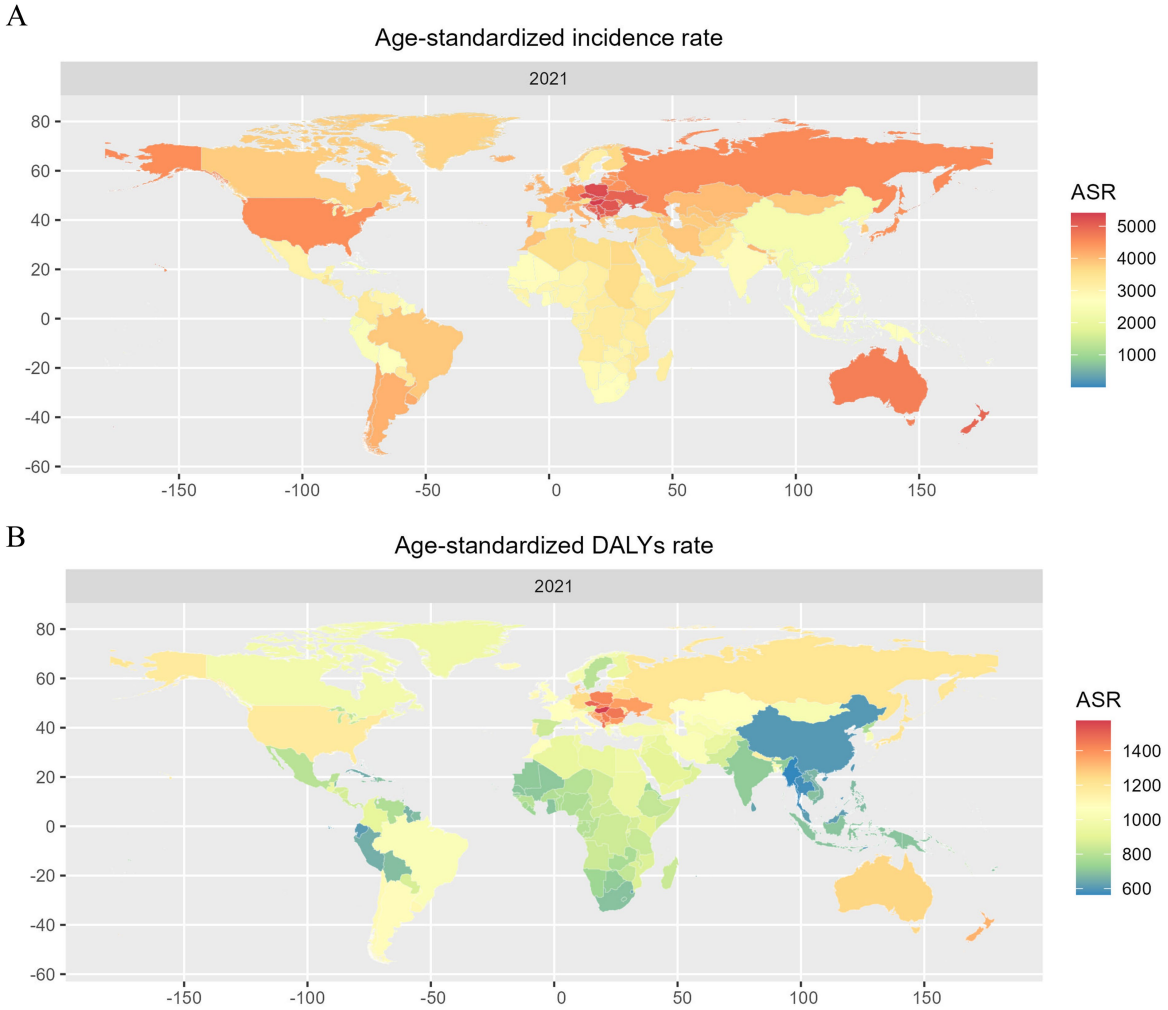
Age-standardized incidence rates **(A)** and DALYs rates **(B)** of LBP for both sexes in 204 countries and territories in 2021. DALYs, disability-adjusted life years.

Across all countries, in 2021, Hungary had the global highest ASIR [5408.81 (95% UI, 4788.39–6085.51) per 100,000 people], whereas Maldives had the lowest [2215.56 (95% UI, 1944.53–2490.89) per 100,000 people] (Figure 4A). The global highest ASDALYsR of LBP [1573.52 (95% UI, 1118.61–2089.53) per 100,000 people] was seen in Hungary, whereas the lowest [564.98 (95% UI, 396.39–750.07) per 100,000 people] was observed in Maldives (Figure 4; Supplementary Tables S2, S3). From 1990 to 2021, China had the largest decrease in ASIR with an EAPC of −0.47% (95% UI, −0.56 to −0.37) and the largest decrease in ASDALYsR with an EAPC of −0.49% (95% UI, −0.6 to −0.39), respectively. While the largest increase in ASIR rise was seen in Sweden with an EAPC of 0.61% (95% UI, 0.46–0.76). The largest increase in ASDALYsR was seen in Pakistan with an EAPC of 0.44% (95% UI, 0.36–0.51) (Figure 4; Supplementary Tables S2, S3).

**Figure 5:**
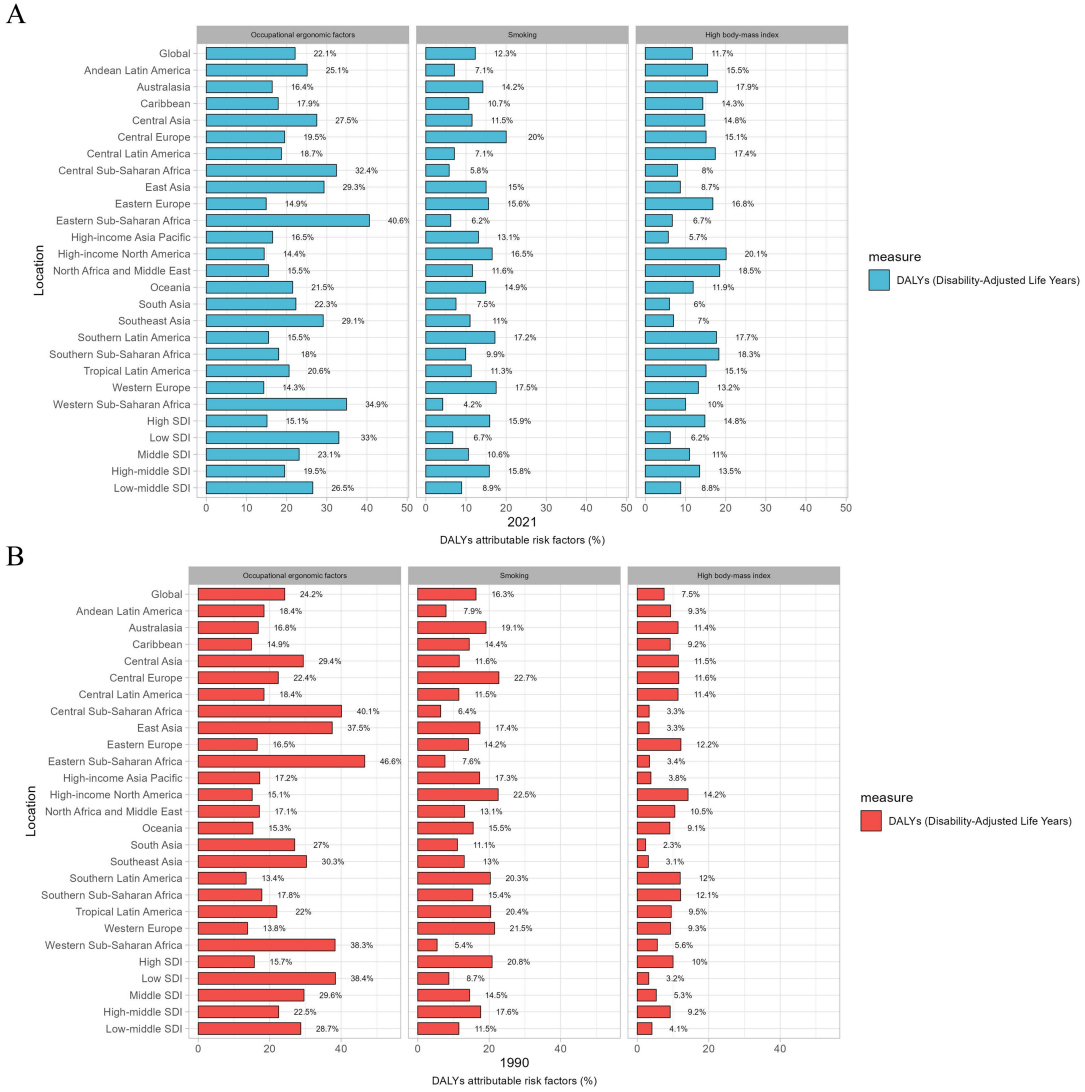
Percentage contribution of major risk factors for both sexes to age-standardized DALYs rate in LBP worldwide, 2021 **(A)** and 1990 **(B)** DALYs, disability adjusted life years; SDI, sociodemographic index.

Among all potential risk factors in GBD 2021, ASDALYsR of LBP worldwide was primarily attributable to occupational ergonomic factors [22.1% (95% UI, 20.3–23.7)], smoking [12.3% (95% UI, 8.2–16.3)], high body mass index (BMI) [11.7% (95% UI, 1.2–22.8)] (Figure 5; Supplementary Table S4). In the 21 GBD regions, the percentage contribution of occupational ergonomic factors and smoking to ASDALYsR of LBP was greater for men than women. Except for the high-income Asia Pacific, the percentage contribution of the high BMI to ASDALYsR of LBP was more influential for women than for men. Across SDI quintiles, occupational ergonomic factors and smoking contributed a greater percentage of ASDALYsR to LBP in men than in women. The effect of the high BMI was reversed. Occupational ergonomic factors, smoking, and high BMI remain the most important attributable risk factors for LBP. Globally, from 1990 to 2021, the percentage contribution of occupational ergonomic factors and smoking to the ASDALYsR of LBP showed a decreasing trend in both sexes, whereas high BMI showed an increasing trend in both sexes.

**Figure 6:**
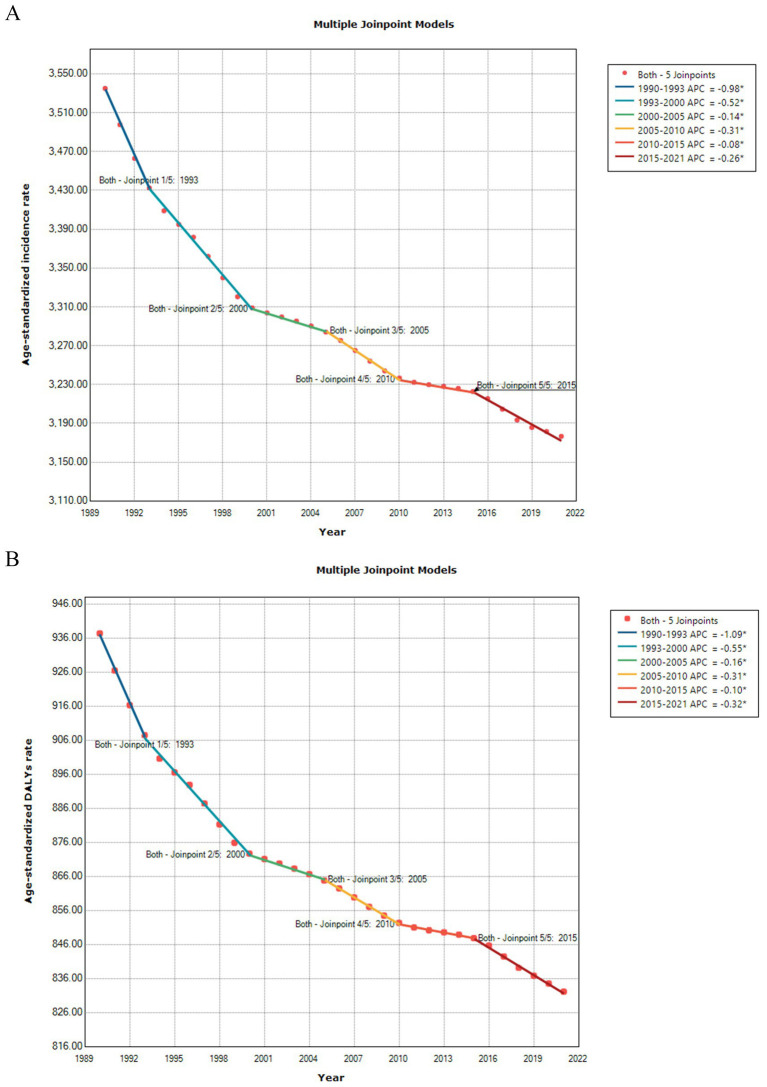
Joinpoint regression model for the age-standardized incidence rate (A) and age-standardized DALYs rate (B) of LBP.

**Figure 7:**
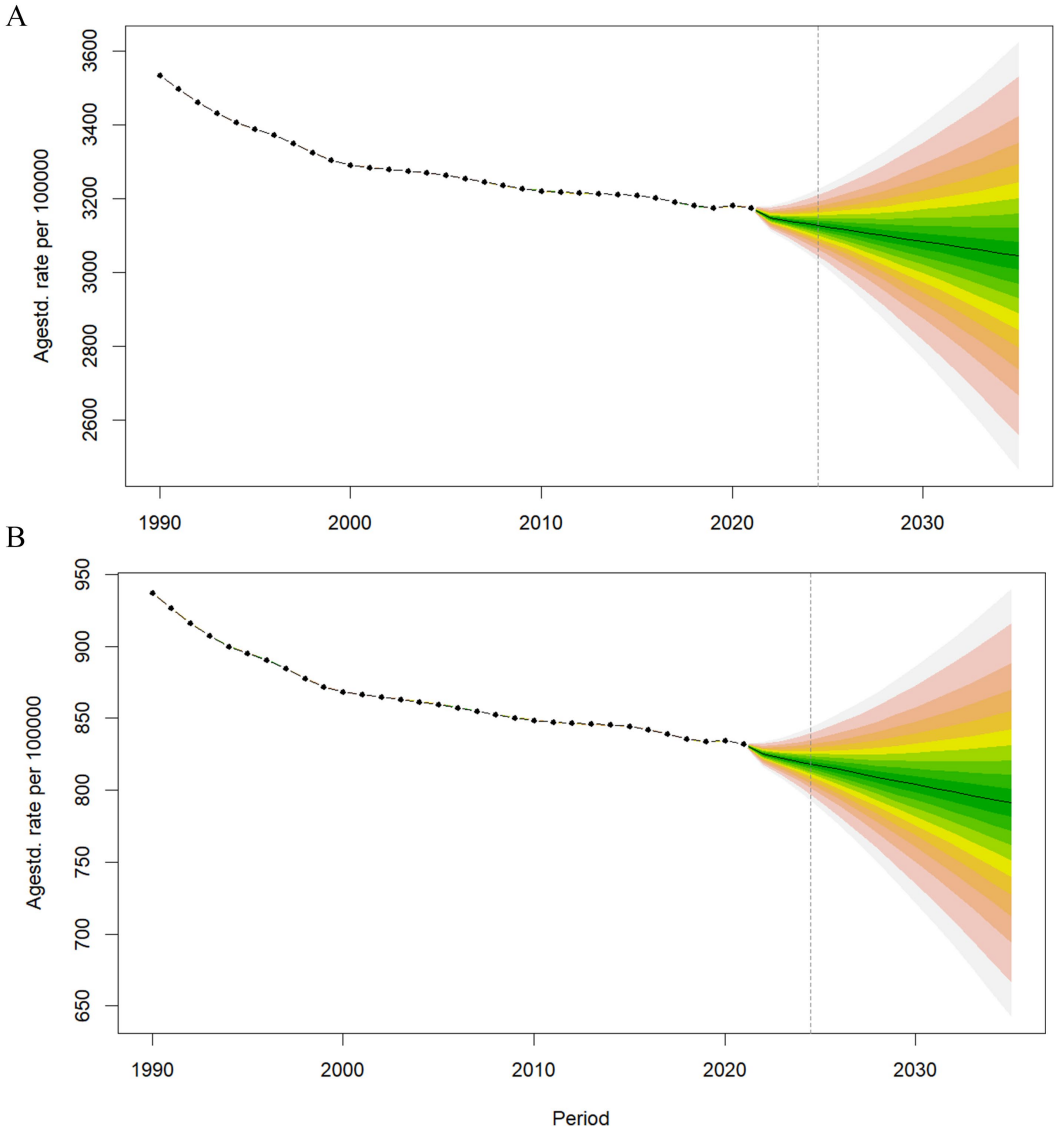
Predicted trends in the age-standardized incidence rates (A) and age-standardized DALY rates (B) of LBP until 2035.

Between 1990 and 2021, the ASIR of LBP showed a decreasing trend worldwide, AAPC = −0.35 (95% CI, −0.37 to −0.32, *p* < 0.001). By dividing the age interval, the largest decrease occurred in the 1990–1993, APC = −0.98 (95% CI, −1.10 to −0.87, *p* < 0.001). In terms of the ASDALYsR of LBP, a decreasing trend was noted during 1990–2021, AAPC = −0.38 (95% CI, −0.40 to −0.36, *p* < 0.001) (Figure 6). The largest decrease in ASDALYsR occurred during 1990–1993, APC = −1.09 (95% CI, −1.19 to −0.99, *p* < 0.001). The trend prediction of ASIR and ASDALYsR is shown in Figure 7.

## Discussion

This study offers detailed information on the disease burden due to LBP, including incidence and DALYs, across 204 countries and territories from 1990 to 2021, using the most recent publicly available model data and methods from GBD study 2021 (6). Furthermore, it provides a comprehensive analysis of the relationship between development levels and LBP burden across regions of varying developmental status worldwide, examines attributable risk factors for LBP, and explores trends in disease progression.

In 2021, LBP accounted for approximately 628.8 million prevalent cases, 266.9 million new incident cases, and 70.2 million DALYs worldwide. Both ASIR and ASDALYsR of LBP showed a decreasing trend from 1990 to 2021 and join point regression showed that ASIR and ASDALYsR of LBP continued to decrease in the past 30 years, although the decreasing trend has slowed in recent years. Predictive analysis suggests that the ASIR and ASDALYsR of LBP are expected to continue to decline by 2035. However, the absolute number of prevalent cases, incidence cases, and number of DALYs increased substantially, LBP remains the leading cause of YLDs, and DALYs increased substantially (6). The highest ASIR and ASDALYsR for LBP were found in Central Europe, with Hungary having the highest ASIR and ASDALYsR among 204 countries, and China has the world’s largest affected population, with 100.1 million individuals suffering from LBP with an ASIR of [2342.46 (95% UI, 2058.05–2639.32) per 100,000 people].

LBP imposes a significant economic and social burden on many countries. In the United States, LBP ranks first among 154 healthcare spending conditions, with approximately $134.5 billion spent in 2016 (14). Recent studies have indicated that the cost of LBP in the United States is approximately $40 billion (or approximately $2,000/patient/year), while it continues to rise (15). In Brazil, the direct cost of surgery for LBP in hospitals funded by the Unified Health System reached R$ (Brazilian currency, Brazilian real) 24,427,238 within 6 years, with an average cost per procedure of R$3,290 (16). A South African study found that the overall average annual cost of direct medical care associated with LBP was $5.4 million, with outpatient care costs accounting for the largest proportion (17). A Japanese study concluded that unemployment in among 1.04 million Japanese male workers are associated with LBP (18).

In this study, women bore a higher burden of LBP than men worldwide in 2021, which is similar to that reported in the GBD studies in 2017 and 2019, but contrasts with the GBD study from 2010 (3–5). The review presents that estrogen changes during menopause may significantly contribute to disk degeneration, leading to LBP. Estrogen receptors in the disk tissue support the potential role of estrogen changes in causing LBP (19). Postmenopausal women also exhibit higher rates of osteoporosis-related spinal fractures and may experience abdominal weight gain during the perimenopausal period, which contributes to the increased incidence of LBP in women, particularly middle-aged women (20). In 2021, both the number of incident cases and DALY cases worldwide increased with age, peaking at the age of 50–54 years, before declining. Global trends in ASRs and ASDALYsR also increased with age, peaking at the age of 80–84 years for both men and women. These data pattern changes are consistent with observations from the GBD study in 2010, 2017, and 2019 (3–5). With increasing age, the incidence of degenerative changes in spinal joints and osteoporosis increases, leading to higher LBP rates among older patients. In addition, aging-related bodily changes increase susceptibility to pain, affect pain tolerance, and reduced ability to recover from injury (21). This vicious cycle explains the age pattern of LBP disease burden worldwide. Thus, early education and disease prevention for female groups, particularly middle-aged perimenopausal women, is necessary. More attention should be paid to the older adults population, and in addition to invasive treatment and oral drugs, measures such as exercise in the early years and psychological intervention in the later years are also necessary.

In 1990–2021, the ASIR of area-level LBP showed a decreasing trend with increasing SDI. However, overall, the ASIR and ASDALYsR of LBP negatively correlated with the SDI when the SDI was >0.8 and nearly positively correlated when the SDI was <0.8. Notably, the ASIR of LBP positively correlated with ASDALYsR and SDI in the GBD 2019 study (3). This downward trend is encouraging, indicating that the LBP burden decreases as socioeconomic development progresses. This may be attributed to advancements in medical technology and improvement in occupational ergonomic factors resulting from industrial upgrading, although the prevalence of obesity and sedentary entertainment increases with economic development. However, inequality between regions remains serious.

Globally, the contribution of high BMI to LBP continues to increase, whereas that of smoking and occupational ergonomic factors continues to decline. A two-sample Mendelian randomization study found a positive association between BMI and LBP (22). The link between obesity and musculoskeletal chronic pain can be explained by both mechanical loading and biochemical mechanisms, where additional body weight imposes greater stress on the spinal joints and back muscles, increasing susceptibility to LBP. Regarding occupational ergonomic factors, a review (23) concluded that sedentary behavior in non-neutral postures at work was associated with LBP in workers. Specifically, bent postures rather than sedentary leisure time are associated with new LBP in nurses, and sitting behavior in call center employees is associated with chronic LBP and functional dysfunction. Several studies have provided evidence of a significant association between smoking and LBP (24, 25). The burden of LBP due to smoking increases over time (26). Smokers tend to report higher pain intensity, require more pain medication, and experience greater effects on their lives because of pain than non-smokers (27). In recent years, the proposed biopsychosocial model suggests that LBP is a dynamic interaction between social, psychological, and biological factors, which can both induce and lead to injury; thus, multidisciplinary combination should be considered when designing treatment options. Although LBP prevention in high-risk groups presents key challenges, early prevention can effectively address the burden of disease and the high healthcare costs associated with treatment and rehabilitation. Therefore, recommendations include enhancing smoking control measures, optimizing the work environment, and encouraging increased physical activity to maintain a healthy BMI. These prevention strategies aim to reduce the incidence of LBP.

This study has some limitations. The effect of COVID-19 on the incidence and burden of LBP was not considered during data analysis and prediction. Reductions in treatment opportunities or even increased mortality due to COVID-19 in older adults affect the LBP burden. Only the main risk factors associated with LBP were analyzed; thus, other potential risk factors contributing to LBP warrant further investigation and discussion. In addition, disease burden may be associated with differences in race, social habits between regions and requires further investigation.

## Conclusion

The global burden of LBP has declined over the last decades, and incidence is expected to remain decreasing by 2035. However, its absolute number remains significantly increased; thus, LBP will remain an important cause of DALYs worldwide. Women and middle-aged populations still need more attention, and inequalities between different SDI quintiles must be taken seriously. Occupational ergonomic factors, high BMI, and smoking remain important risk factors for LBP. Effective preventive and intervention measures are needed to further reduce the burden of LBP.

## Data Availability

The raw data supporting the conclusions of this article will be made available by the authors, without undue reservation.

## Footnotes

^1^http://ghdx.healthdata.org/gbd-results-tool

^2^https://surveillance.cancer.gov/joinpoint/download

## References

[1] GBD 2021 Low Back Pain Collaborators. Global, regional, and national burden of low back pain, 1990-2020, its attributable risk factors, and projections to 2050: a systematic analysis of the global burden of disease study 2021. Lancet Rheumatol. (2023) 5:e316–29. doi: 10.1016/S2665-9913(23)00098-X 37273833 PMC10234592

[2] Knezevic NN Candido KD Vlaeyen JWS Van Zundert J Cohen SP. Low back pain. Lancet. (2021) 398:78–92. doi: 10.1016/S0140-6736(21)00733-9 34115979

[3] Chen S Chen M Wu X Lin S Tao C Cao H. Global, regional and national burden of low back pain 1990-2019: a systematic analysis of the global burden of disease study 2019. J Orthop Translat. (2022) 32:49–58. doi: 10.1016/j.jot.2021.07.005, PMID: 34934626 PMC8639804

[4] Hoy D March L Brooks P Blyth F Woolf A Bain C. The global burden of low back pain: estimates from the global burden of disease 2010 study. Ann Rheum Dis. (2014) 73:968–74. doi: 10.1136/annrheumdis-2013-204428 24665116

[5] Wu A March L Zheng X Huang J Wang X Zhao J. Global low back pain prevalence and years lived with disability from 1990 to 2017: estimates from the global burden of disease study 2017. Ann Transl Med. (2020) 8:299. doi: 10.21037/atm.2020.02.175, PMID: 32355743 PMC7186678

[6] GBD 2021 Diseases and Injuries Collaborators. Global incidence, prevalence, years lived with disability (YLDs), disability-adjusted life-years (DALYs), and healthy life expectancy (HALE) for 371 diseases and injuries in 204 countries and territories and 811 subnational locations, 1990-2021: a systematic analysis for the global burden of disease study 2021. Lancet. (2024) 403:2133–61. doi: 10.1016/S0140-6736(24)00757-8, PMID: 38642570 PMC11122111

[7] Liu Z Jiang Y Yuan H Fang Q Cai N Suo C. The trends in incidence of primary liver cancer caused by specific etiologies: results from the global burden of disease study 2016 and implications for liver cancer prevention. J Hepatol. (2019) 70:674–83. doi: 10.1016/j.jhep.2018.12.001, PMID: 30543829

[8] Gao S Yang WS Bray F Va P Zhang W Gao J. Declining rates of hepatocellular carcinoma in urban Shanghai: incidence trends in 1976-2005. Eur J Epidemiol. (2012) 27:39–46. doi: 10.1007/s10654-011-9636-8, PMID: 22160277 PMC5477645

[9] Cronin KA Ries LA Edwards BK. The surveillance, epidemiology, and end results (SEER) program of the National Cancer Institute. Cancer. (2014) 120:3755–7. doi: 10.1002/cncr.29049 25412387

[10] Cayuela A Rodríguez-Domínguez S Jara-Palomares L Otero-Candelera R López-Campos JL Vigil E. Gender differences in lung cancer mortality trends in Andalusia 1975-2008: a joinpoint regression analysis. Med Oncol. (2012) 29:1593–8. doi: 10.1007/s12032-011-0007-9, PMID: 21678025

[11] Ebrahimi Kalan M Jebai R Li W Gautam P Osibogun O Alqahtani MM. High on hookah: smoking marijuana from a hookah among adults in the United States, population assessment of tobacco and health study, 2015-2019. Subst Use Misuse. (2023) 58:657–65. doi: 10.1080/10826084.2023.2177966, PMID: 36786640 PMC10069405

[12] GBD 2021 Demographics Collaborators. Global age-sex-specific mortality, Life expectancy, and population estimates in 204 countries and territories and 811 subnational locations, 1950-2021, and the impact of the COVID-19 pandemic: a comprehensive demographic analysis for the global burden of disease study 2021. Lancet. (2024) 403:1989–2056. doi: 10.1016/S0140-6736(24)00476-8 38484753 PMC11126395

[13] Vollset SE Goren E Yuan CW Cao J Smith AE Hsiao T. Fertility, mortality, migration, and population scenarios for 195 countries and territories from 2017 to 2100: a forecasting analysis for the global burden of disease study. Lancet. (2020) 396:1285–306. doi: 10.1016/S0140-6736(20)30677-2, PMID: 32679112 PMC7561721

[14] Dieleman JL Cao J Chapin A Chen C Li Z Liu A. US health care spending by payer and health condition, 1996-2016. JAMA. (2020) 323:863–84. doi: 10.1001/jama.2020.0734, PMID: 32125402 PMC7054840

[15] Chang D Lui A Matsoyan A Safaee MM Aryan H Ames C. Comparative review of the socioeconomic burden of lower Back pain in the United States and globally. Neurospine. (2024) 21:487–501. doi: 10.14245/ns.2448372.186, PMID: 38955526 PMC11224735

[16] Mendonça AG Oliveira VC Fonseca LS Oliveira MX. Direct costs of low back pain in hospitals financed by the unified health system. Rev Pesqui Fisioter. (2021) 11:1–9. doi: 10.17267/2238-2704rpf.v11i1.3438

[17] Kahere M Ngcamphalala C Östensson E Ginindza T. The economic burden of low back pain in KwaZulu-Natal, South Africa: a prevalence-based cost-of-illness analysis from the healthcare provider’s perspective. PLoS One. (2022) 17:e0263204. doi: 10.1371/journal.pone.0263204, PMID: 36227919 PMC9560048

[18] Tomioka K Kitahara T Shima M Saeki K. Fraction and number of unemployed associated with self-reported low Back pain: a nation-wide cross-sectional study in Japan. Int J Environ Res Public Health. (2021) 18:760. doi: 10.3390/ijerph182010760 34682501 PMC8536185

[19] Pang H Chen S Klyne DM Harrich D Ding W Yang S. Low back pain and osteoarthritis pain: a perspective of estrogen. Bone Res. (2023) 11:42. doi: 10.1038/s41413-023-00280-x, PMID: 37542028 PMC10403578

[20] Wang YXJ. Menopause as a potential cause for higher prevalence of low back pain in women than in age-matched men. J Orthop Translat. (2017) 8:1–4. doi: 10.1016/j.jot.2016.05.012, PMID: 30035087 PMC5987020

[21] Mullins S Hosseini F Gibson W Thake M. Physiological changes from ageing regarding pain perception and its impact on pain management for older adults. Clin Med. (2022) 22:307–10. doi: 10.7861/clinmed.22.4.phys, PMID: 35882493 PMC9345212

[22] Chen X Tang H Lin J Zeng R. Causal relationships of obesity on musculoskeletal chronic pain: a two-sample Mendelian randomization study. Front Endocrinol. (2022) 13:971997. doi: 10.3389/fendo.2022.971997, PMID: 36082069 PMC9445165

[23] Baradaran Mahdavi S Riahi R Vahdatpour B Kelishadi R. Association between sedentary behavior and low back pain; a systematic review and meta-analysis. Health Promot Perspect. (2021) 11:393–410. doi: 10.34172/hpp.2021.50, PMID: 35079583 PMC8767074

[24] Hashimoto Y Matsudaira K Sawada SS Gando Y Kawakami R Kinugawa C. Objectively measured physical activity and low back pain in Japanese men. J Phys Act Health. (2018) 15:417–22. doi: 10.1123/jpah.2017-0085, PMID: 29542388

[25] Schembri E Massalha V Camilleri L Lungaro-Mifsud S. Is chronic low back pain and radicular neuropathic pain associated with smoking and a higher nicotine dependence? A cross-sectional study using the DN4 and the Fagerström test for nicotine dependence. Agri. (2021) 33:155–67. doi: 10.14744/agri.2021.79836, PMID: 34318914

[26] Fan Z Li W Wang Z. Change trend analysis of the disease burden of low back pain and its risk factors in China from 1990 to 2019. Chinese J Dis Control Prev. (2023) 27:807–13. doi: 10.16462/j.cnki.zhjbkz.2023.07.011

[27] Khan JS Hah JM Mackey SC. Effects of smoking on patients with chronic pain: a propensity-weighted analysis on the collaborative health outcomes information registry. Pain. (2019) 160:2374–9. doi: 10.1097/j.pain.0000000000001631, PMID: 31149975 PMC6768701

