# Supplementary material for: Global burden of low back pain and its attributable risk factors from 1990 to 2021: a comprehensive analysis from the global burden of disease study 2021
Source: Front Public Health. 2024 Nov 13;12:1480779. doi: 10.3389/fpubh.2024.1480779 (PMC11598917; doi:10.3389/fpubh.2024.1480779)
Supplement: Supplementary file 1 [file Table_1.docx]

Table S1. Prevalence of Low back pain in 1990 and 2021 for both sexes in regions, with EAPC from 1990 to 2021.

| Location | Num 1990 | ASR 1990 | Num 2021 | ASR 2021 | EAPC CI |
| --- | --- | --- | --- | --- | --- |
| Global | 386731361 (341581662 to 434164620) | 8391.58 (7381.14 to 9367.39) | 628838475 (551834407 to 700881341) | 7463.13 (6575.68 to 8321.8) | -0.32% (-0.35 to -0.28) |
| High-middle SDI | 93734190 (82340884 to 105198177) | 8897.05 (7815.47 to 9917.76) | 128827069 (113031393 to 144240276) | 7631.54 (6693.46 to 8509.81) | -0.43% (-0.47 to -0.38) |
| High-middle SDI | 93734190 (82340884 to 105198177) | 8897.05 (7815.47 to 9917.76) | 128827069 (113031393 to 144240276) | 7631.54 (6693.46 to 8509.81) | -0.43% (-0.47 to -0.38) |
| High SDI | 105597928 (94355803 to 117381974) | 10585.74 (9467.02 to 11806.57) | 143792047 (129652681 to 157076124) | 9783.64 (8876.62 to 10734.23) | -0.19% (-0.21 to -0.16) |
| Low-middle SDI | 65025917 (57621208 to 73772912) | 7762.41 (6800.92 to 8691.74) | 126191233 (110548447 to 142904183) | 7317.71 (6386.63 to 8227.13) | -0.18% (-0.23 to -0.14) |
| Low SDI | 25218245 (22268893 to 28454461) | 7841.48 (6870.11 to 8781.4) | 55999862 (49426590 to 63405672) | 7364.87 (6427.17 to 8255.87) | -0.21% (-0.23 to -0.19) |
| Middle SDI | 96691431 (85105762 to 109488201) | 7044.2 (6156.41 to 7905.07) | 173417025 (150668277 to 195125730) | 6421.04 (5616.75 to 7199.09) | -0.2% (-0.25 to -0.15) |
| Andean Latin America | 1640636 (1443039 to 1863568) | 5748.76 (5034.13 to 6474.59) | 3735038 (3274646 to 4209857) | 5769.81 (5050.62 to 6502.89) | 0.03% (0.01 to 0.06) |
| Australasia | 2728928 (2438871 to 3043336) | 12314.65 (11042.6 to 13718.62) | 4389558 (3843185 to 4905996) | 11327.03 (9980.82 to 12746.38) | -0.19% (-0.22 to -0.17) |
| Caribbean | 1873836 (1653012 to 2118352) | 6092.39 (5347.57 to 6854.23) | 3086535 (2735228 to 3457070) | 6006.95 (5348.69 to 6726.06) | -0.01% (-0.02 to 0) |
| Central Asia | 5123333 (4534991 to 5736765) | 9298.22 (8196.51 to 10320.92) | 8445849 (7373427 to 9503998) | 9188.48 (8032.34 to 10254.54) | -0.03% (-0.04 to -0.02) |
| Central Europe | 18424682 (16237814 to 20534270) | 13198.96 (11644.23 to 14728.93) | 20610737 (18182273 to 22911381) | 12831.04 (11293.99 to 14267.18) | -0.1% (-0.11 to -0.09) |
| Central Latin America | 9231074 (8138286 to 10600871) | 7394.45 (6490.72 to 8348.32) | 19673938 (17310811 to 22160905) | 7487.05 (6597.82 to 8413.59) | 0.04% (0 to 0.09) |
| Central Sub-Saharan Africa | 2666476 (2333799 to 3016266) | 7872.21 (6875.11 to 8803.69) | 6759109 (5963457 to 7709322) | 7619.29 (6670.28 to 8565.24) | -0.13% (-0.16 to -0.1) |
| East Asia | 70972106 (61584149 to 80733977) | 6652.23 (5797.61 to 7476.49) | 105135609 (91895083 to 118365184) | 5418.74 (4746.47 to 6045.66) | -0.47% (-0.57 to -0.36) |
| Eastern Europe | 30221827 (26656521 to 33743437) | 11653.11 (10264.38 to 12960.92) | 31818730 (28084336 to 35390254) | 11189.88 (9858.28 to 12447.24) | -0.07% (-0.09 to -0.05) |
| Eastern Sub-Saharan Africa | 8853965 (7817977 to 10027008) | 7890 (6905.07 to 8826.62) | 20671419 (18268068 to 23441517) | 7607.2 (6640.3 to 8512.38) | -0.13% (-0.14 to -0.12) |
| High-income Asia Pacific | 21730692 (19102521 to 24328946) | 11083.01 (9779.81 to 12436.64) | 27054791 (23812392 to 30032356) | 10041.05 (8876.21 to 11287.71) | -0.27% (-0.29 to -0.25) |
| High-income North America | 35237734 (31463631 to 39080410) | 11238.41 (10024.5 to 12532.94) | 49456310 (46021985 to 52598499) | 10475.84 (9747.66 to 11177.32) | -0.07% (-0.13 to -0.02) |
| North Africa and Middle East | 22399030 (19902203 to 25305533) | 8937.83 (7994.23 to 9965.56) | 50601960 (44810213 to 57246491) | 8713.52 (7713 to 9767.15) | -0.06% (-0.08 to -0.05) |
| Oceania | 282858 (248890 to 321690) | 6380.65 (5600.75 to 7119.26) | 674940 (591101 to 768285) | 6322.16 (5498.31 to 7093.65) | -0.01% (-0.02 to 0.01) |
| South Asia | 61510959 (54295998 to 69663661) | 7735.78 (6764.27 to 8668.52) | 119421340 (104121215 to 135119660) | 6919.73 (6023.41 to 7778.72) | -0.36% (-0.45 to -0.26) |
| Southeast Asia | 20564245 (18259667 to 23269347) | 5936.27 (5227.83 to 6631.53) | 42068815 (36528833 to 47513123) | 5859.18 (5118.41 to 6584.8) | -0.02% (-0.03 to -0.01) |
| Southern Latin America | 4668528 (4103323 to 5247668) | 9770.4 (8585.81 to 10945.33) | 7546942 (6657533 to 8456318) | 9741.14 (8600.77 to 10945.72) | -0.02% (-0.06 to 0.02) |
| Southern Sub-Saharan Africa | 2452713 (2151707 to 2750769) | 6840.43 (5957.72 to 7647.28) | 4577210 (3987735 to 5172963) | 6510.35 (5661.38 to 7303.04) | -0.13% (-0.14 to -0.11) |
| Tropical Latin America | 11347302 (10034300 to 12883622) | 9009.3 (7876.51 to 10155.83) | 23454673 (20450315 to 26355944) | 9303.75 (8187.67 to 10456.01) | 0.07% (0.04 to 0.1) |
| Western Europe | 46066166 (41107299 to 50853208) | 9854.31 (8811 to 10969.29) | 58122167 (51179306 to 64598752) | 9533.04 (8439.69 to 10690.62) | -0.07% (-0.08 to -0.06) |
| Western Sub-Saharan Africa | 8734271 (7720212 to 9871622) | 7094.79 (6213.33 to 7933.42) | 21532804 (18994996 to 24407091) | 6911.71 (6038.99 to 7750.39) | -0.1% (-0.13 to -0.06) |
