# Supplementary material for: Global burden of low back pain and its attributable risk factors from 1990 to 2021: a comprehensive analysis from the global burden of disease study 2021
Source: Front Public Health. 2024 Nov 13;12:1480779. doi: 10.3389/fpubh.2024.1480779 (PMC11598917; doi:10.3389/fpubh.2024.1480779)
Supplement: Supplementary file 2 [file Table_2.docx]

Table S2. Incidence of Low back pain in 1990 and 2021 for both sexes in 204 countries, with EAPC from 1990 to 2021.

| Location | Num 1990 | ASR 1990 | Num 2021 | ASR 2021 | EAPC CI |
| --- | --- | --- | --- | --- | --- |
| Afghanistan | 283230 (248804 to 317563) | 3628.83 (3219.91 to 4103.38) | 763858 (660141 to 866459) | 3648.4 (3213.24 to 4123.2) | 0.01% (-0.01 to 0.02) |
| Albania | 144445 (127283 to 164169) | 5166.37 (4575.53 to 5815.59) | 173589 (151725 to 193704) | 5171.35 (4567.64 to 5848.59) | 0% (-0.01 to 0) |
| Algeria | 680749 (593215 to 773819) | 3599.51 (3162.44 to 4096.98) | 1513180 (1336387 to 1706644) | 3574.04 (3156.27 to 4026.17) | -0.04% (-0.05 to -0.02) |
| American Samoa | 977 (856 to 1121) | 2814.56 (2461.9 to 3186.64) | 1399 (1214 to 1587) | 2770.28 (2426.17 to 3132.16) | -0.04% (-0.06 to -0.03) |
| Andorra | 2463 (2164 to 2791) | 4062.48 (3585.81 to 4593.23) | 4617 (4022 to 5187) | 3937.23 (3487.54 to 4430.03) | -0.07% (-0.08 to -0.06) |
| Angola | 220228 (191524 to 250373) | 3376.27 (2960.29 to 3826.45) | 670584 (585007 to 768633) | 3256.05 (2874.95 to 3682.81) | -0.14% (-0.17 to -0.11) |
| Antigua and Barbuda | 1468 (1296 to 1659) | 2617.54 (2290.5 to 2949.26) | 2704 (2352 to 3071) | 2603.26 (2283.6 to 2945.78) | -0.02% (-0.04 to -0.01) |
| Argentina | 1350405 (1195288 to 1526846) | 4172.45 (3697.91 to 4719.77) | 2083325 (1842225 to 2339082) | 4121.75 (3641.43 to 4638.04) | -0.02% (-0.06 to 0.02) |
| Armenia | 128681 (113142 to 145461) | 4127.39 (3619.41 to 4653.58) | 152558 (133728 to 173186) | 4132.29 (3659.25 to 4672.38) | 0.02% (0.02 to 0.03) |
| Australia | 923294 (822051 to 1045980) | 5025.48 (4495.85 to 5709.17) | 1482484 (1308569 to 1656553) | 4665.14 (4129.02 to 5233.03) | -0.16% (-0.19 to -0.14) |
| Austria | 360907 (318378 to 402755) | 3821.97 (3380.36 to 4293.43) | 439080 (381510 to 494633) | 3575.91 (3137.65 to 3999.45) | -0.07% (-0.12 to -0.02) |
| Azerbaijan | 237870 (209614 to 270410) | 3903.25 (3441.23 to 4393.8) | 444581 (389986 to 509756) | 3925.38 (3472.61 to 4427.53) | 0.05% (0.04 to 0.06) |
| Bahamas | 5747 (5016 to 6504) | 2623.74 (2290.78 to 2955.25) | 11202 (9844 to 12754) | 2612.99 (2296.92 to 2954.23) | -0.03% (-0.04 to -0.02) |
| Bahrain | 14329 (12279 to 16607) | 3500.43 (3093.07 to 3973.9) | 53328 (46125 to 61308) | 3452.89 (3036.39 to 3866.39) | -0.05% (-0.07 to -0.04) |
| Bangladesh | 2852974 (2542304 to 3240359) | 3893.01 (3468.87 to 4409.64) | 5855108 (5159977 to 6637268) | 3696.16 (3251.54 to 4180.37) | -0.12% (-0.17 to -0.08) |
| Barbados | 7053 (6255 to 7942) | 2658.48 (2343.34 to 3001.85) | 10311 (9029 to 11637) | 2628.71 (2307.02 to 2963.17) | -0.03% (-0.04 to -0.03) |
| Belarus | 527737 (464315 to 595739) | 4477.18 (3939.87 to 5053.86) | 559125 (490635 to 628859) | 4457.88 (3949.39 to 5007.89) | 0% (-0.01 to 0) |
| Belgium | 505640 (446320 to 568645) | 4175.31 (3706.01 to 4731.36) | 615778 (540304 to 689454) | 4062.1 (3591.99 to 4569.05) | -0.07% (-0.08 to -0.06) |
| Belize | 3534 (3094 to 3979) | 2653.6 (2329.38 to 3001.37) | 10499 (9214 to 11860) | 2663.04 (2334.58 to 3007.91) | 0.03% (0.02 to 0.04) |
| Benin | 92100 (81080 to 104443) | 3130.54 (2746.19 to 3540.63) | 257773 (225982 to 292108) | 2991.77 (2627.95 to 3366.14) | -0.15% (-0.19 to -0.12) |
| Bermuda | 1756 (1538 to 2008) | 2695.33 (2369.62 to 3046.84) | 2423 (2102 to 2756) | 2663.36 (2342.45 to 3034.71) | -0.04% (-0.05 to -0.04) |
| Bhutan | 15323 (13434 to 17318) | 3648.19 (3219.31 to 4095.51) | 26195 (23057 to 29894) | 3578.73 (3161.51 to 4052.08) | -0.06% (-0.06 to -0.05) |
| Bolivia (Plurinational State of) | 123969 (108627 to 140053) | 2638.84 (2305.86 to 2964.44) | 292923 (258065 to 333401) | 2667.58 (2353.16 to 3037.04) | 0.02% (-0.02 to 0.05) |
| Bosnia and Herzegovina | 225751 (199134 to 255132) | 4903.52 (4340.48 to 5493.18) | 223106 (196219 to 250665) | 4991.8 (4457.19 to 5623.81) | 0.05% (0.04 to 0.07) |
| Botswana | 23715 (20723 to 26571) | 2844.47 (2497.47 to 3217.79) | 57601 (50175 to 66036) | 2813.18 (2454.09 to 3178.56) | -0.03% (-0.04 to -0.01) |
| Brazil | 4763353 (4167395 to 5431370) | 3794.63 (3343.85 to 4291.17) | 9426567 (8304552 to 10610219) | 3873.61 (3416.6 to 4355.03) | 0.04% (0.02 to 0.06) |
| Brunei Darussalam | 8154 (7033 to 9297) | 3891.56 (3446.93 to 4357.9) | 17649 (15449 to 20151) | 3730.92 (3291.22 to 4221.97) | -0.11% (-0.13 to -0.09) |
| Bulgaria | 533760 (468894 to 602590) | 5093.31 (4514.99 to 5792.57) | 478411 (418532 to 539349) | 5013.54 (4412.83 to 5638.08) | -0.05% (-0.05 to -0.04) |
| Burkina Faso | 185432 (162443 to 207181) | 3101.31 (2740.03 to 3465.15) | 440766 (385102 to 500134) | 3003.88 (2632.77 to 3408.28) | -0.15% (-0.18 to -0.12) |
| Burundi | 117464 (102756 to 133607) | 3376.7 (2971.92 to 3836.16) | 272446 (237776 to 309421) | 3206.63 (2806.25 to 3603.47) | -0.2% (-0.22 to -0.19) |
| C?te d'Ivoire | 235578 (206609 to 267183) | 3160.52 (2782.23 to 3560.46) | 585762 (514674 to 670846) | 3042.66 (2665.81 to 3418.73) | -0.12% (-0.17 to -0.08) |
| Cabo Verde | 7435 (6529 to 8431) | 2993.49 (2626.91 to 3396.25) | 15110 (13159 to 17110) | 2812.93 (2456.78 to 3172.25) | -0.22% (-0.26 to -0.18) |
| Cambodia | 189340 (165445 to 215200) | 2819.37 (2475.89 to 3183.98) | 401347 (349565 to 455754) | 2626.13 (2304.62 to 2967.77) | -0.24% (-0.25 to -0.23) |
| Cameroon | 215116 (189284 to 243504) | 3212.41 (2819.4 to 3597.36) | 655123 (580004 to 746966) | 3062.43 (2700.48 to 3470.95) | -0.18% (-0.22 to -0.15) |
| Canada | 1262603 (1113066 to 1432253) | 4147.99 (3666.56 to 4720.44) | 1842152 (1614666 to 2066842) | 3816.85 (3352.25 to 4313.39) | -0.18% (-0.21 to -0.15) |
| Central African Republic | 59409 (52157 to 67437) | 3334.42 (2941.47 to 3742.03) | 123057 (108266 to 139601) | 3282.66 (2912.09 to 3717.92) | -0.07% (-0.08 to -0.05) |
| Chad | 133116 (118007 to 150086) | 3446.82 (3035.84 to 3916.04) | 327234 (287064 to 371603) | 3201.86 (2811.17 to 3624.86) | -0.22% (-0.29 to -0.15) |
| Chile | 516950 (455193 to 582712) | 4213.32 (3733.46 to 4751.91) | 920387 (810575 to 1034465) | 4166.18 (3690.56 to 4690.88) | -0.03% (-0.07 to 0) |
| China | 29843970 (26065824 to 34012369) | 2859.38 (2508.61 to 3225.53) | 43374995 (37494376 to 49159184) | 2342.46 (2058.05 to 2639.32) | -0.47% (-0.56 to -0.37) |
| Colombia | 903584 (789482 to 1025737) | 3383.77 (2979.52 to 3809.18) | 1808891 (1606166 to 2042895) | 3379.93 (3001.54 to 3827.31) | 0.02% (0 to 0.03) |
| Comoros | 9119 (8013 to 10231) | 3088.52 (2738.4 to 3466.15) | 19459 (17069 to 22015) | 3086.63 (2699.23 to 3485.88) | -0.05% (-0.07 to -0.03) |
| Congo | 50455 (44436 to 56888) | 3171.34 (2812.83 to 3570.09) | 132027 (115674 to 150889) | 3135.91 (2766.23 to 3532.56) | -0.06% (-0.08 to -0.05) |
| Cook Islands | 432 (376 to 489) | 2769.7 (2427.84 to 3137.24) | 609 (531 to 688) | 2817.3 (2478.52 to 3189.97) | 0.09% (0.08 to 0.1) |
| Costa Rica | 81358 (70986 to 92768) | 3270.2 (2877.3 to 3750.16) | 168302 (147931 to 189430) | 3194.58 (2822.58 to 3589.83) | -0.06% (-0.07 to -0.06) |
| Croatia | 281153 (249276 to 317681) | 4996.03 (4461.57 to 5628.07) | 288442 (250497 to 324313) | 4920.53 (4339.57 to 5551.98) | -0.03% (-0.07 to 0) |
| Cuba | 292053 (255511 to 330655) | 2661.94 (2329.27 to 3008.08) | 378545 (336935 to 421999) | 2532.31 (2269.46 to 2801.52) | -0.07% (-0.1 to -0.05) |
| Cyprus | 33568 (29442 to 37793) | 4113.71 (3607.29 to 4626.26) | 68374 (59812 to 77134) | 4030.86 (3557.5 to 4573.67) | -0.06% (-0.07 to -0.05) |
| Czechia | 641839 (564408 to 724080) | 5370.63 (4742.2 to 6042.4) | 757515 (660732 to 856685) | 5240.01 (4603.17 to 5944.22) | -0.08% (-0.09 to -0.08) |
| Democratic People's Republic of Korea | 586649 (511548 to 664668) | 3090.73 (2693.81 to 3477.81) | 919260 (799725 to 1045842) | 2897.39 (2550.15 to 3263.16) | -0.23% (-0.25 to -0.21) |
| Democratic Republic of the Congo | 819174 (713904 to 928844) | 3376.22 (2948.11 to 3807.59) | 1996433 (1761131 to 2272214) | 3272.37 (2886.54 to 3697.98) | -0.12% (-0.15 to -0.09) |
| Denmark | 272907 (236380 to 312312) | 4340.92 (3763.84 to 4983.61) | 302691 (254278 to 354091) | 3907.44 (3319.52 to 4599.9) | -0.48% (-0.56 to -0.4) |
| Djibouti | 8065 (7028 to 9143) | 3090.16 (2722.52 to 3472.19) | 29734 (26266 to 34110) | 2940.52 (2581.14 to 3320.21) | -0.19% (-0.2 to -0.18) |
| Dominica | 1722 (1510 to 1931) | 2714.77 (2382.02 to 3062.64) | 2030 (1781 to 2301) | 2635.05 (2322.98 to 2987.04) | -0.12% (-0.13 to -0.11) |
| Dominican Republic | 144783 (127014 to 163024) | 2614.82 (2306.61 to 2931.52) | 291607 (256609 to 331313) | 2672 (2351.56 to 3026.82) | 0.05% (0.03 to 0.07) |
| Ecuador | 194732 (171858 to 218099) | 2535.85 (2245.48 to 2844.29) | 428993 (379676 to 480955) | 2430.22 (2150.88 to 2727.19) | -0.15% (-0.23 to -0.07) |
| Egypt | 1531588 (1337336 to 1741248) | 3594.42 (3190.26 to 4062.13) | 3317200 (2880493 to 3762644) | 3671.83 (3224.33 to 4137.74) | 0.07% (0.05 to 0.1) |
| El Salvador | 132194 (114870 to 149505) | 3218.45 (2816.2 to 3634.58) | 207220 (183196 to 235931) | 3265.02 (2888.89 to 3724.13) | 0.08% (0.07 to 0.09) |
| Equatorial Guinea | 9236 (8134 to 10458) | 3336.86 (2943.04 to 3743.7) | 32004 (28079 to 36364) | 3179.37 (2808.3 to 3585.74) | -0.18% (-0.2 to -0.16) |
| Eritrea | 59919 (52460 to 67791) | 2971.95 (2612.74 to 3328.39) | 137184 (119912 to 155942) | 2970.03 (2606.56 to 3337.55) | 0.03% (0.01 to 0.04) |
| Estonia | 80756 (71582 to 90519) | 4432.32 (3952.91 to 4943.6) | 80918 (71101 to 91316) | 4409.91 (3888.54 to 4941.14) | 0% (-0.01 to 0.02) |
| Eswatini | 12279 (10740 to 13741) | 2633.68 (2329.51 to 2949.08) | 21763 (19117 to 24709) | 2591.16 (2278.19 to 2909.88) | -0.09% (-0.12 to -0.06) |
| Ethiopia | 1052933 (918645 to 1188316) | 3413.41 (3003.48 to 3830.14) | 2310711 (2017340 to 2628932) | 3184.86 (2799.69 to 3597.67) | -0.23% (-0.24 to -0.22) |
| Fiji | 15794 (13789 to 18058) | 2789.97 (2447.3 to 3160.44) | 23876 (20886 to 27151) | 2708.91 (2371.3 to 3045.83) | -0.09% (-0.1 to -0.09) |
| Finland | 231204 (204155 to 259034) | 3834.44 (3411.72 to 4283.88) | 283434 (248428 to 320363) | 3664.55 (3234 to 4088.35) | -0.1% (-0.12 to -0.08) |
| France | 2750833 (2443859 to 3074845) | 4105.18 (3654.69 to 4613.02) | 3561233 (3151402 to 3977792) | 4059.39 (3594.93 to 4550.5) | -0.04% (-0.05 to -0.02) |
| Gabon | 22529 (19651 to 25290) | 3141.76 (2768.61 to 3553.04) | 45379 (39868 to 51709) | 3117.89 (2741.95 to 3513.15) | -0.03% (-0.04 to -0.02) |
| Gambia | 17471 (15254 to 19794) | 2923.58 (2556.71 to 3303.35) | 45638 (40159 to 51457) | 2839.33 (2480.86 to 3198.53) | -0.11% (-0.15 to -0.07) |
| Georgia | 222844 (196652 to 250922) | 3747.78 (3320.39 to 4199.35) | 172356 (151079 to 194886) | 3705.43 (3276.22 to 4183.33) | -0.11% (-0.14 to -0.07) |
| Germany | 4663145 (4097906 to 5296776) | 4711.39 (4185.1 to 5374.12) | 5280955 (4649960 to 5946989) | 4479.42 (3974.85 to 5045.12) | -0.1% (-0.12 to -0.08) |
| Ghana | 281544 (248696 to 318542) | 2896.17 (2574.57 to 3228.31) | 704425 (623088 to 790114) | 2752.73 (2452.62 to 3085.33) | -0.19% (-0.22 to -0.16) |
| Greece | 491501 (435135 to 548813) | 3952.39 (3505.27 to 4404.25) | 560188 (490340 to 631150) | 3888.68 (3413.09 to 4415.72) | -0.07% (-0.09 to -0.04) |
| Greenland | 2104 (1829 to 2428) | 3968.4 (3490.22 to 4518.96) | 2414 (2082 to 2743) | 3748.66 (3285.91 to 4249.96) | -0.11% (-0.15 to -0.08) |
| Grenada | 1940 (1720 to 2184) | 2662.97 (2332.81 to 3007.29) | 2974 (2572 to 3361) | 2623.42 (2290.17 to 2949.02) | -0.04% (-0.05 to -0.03) |
| Guam | 3113 (2718 to 3558) | 2751.46 (2418.11 to 3125.36) | 5037 (4393 to 5713) | 2727.98 (2400.36 to 3096.88) | -0.02% (-0.03 to 0) |
| Guatemala | 208115 (183619 to 236915) | 3625.92 (3206.64 to 4102.08) | 495472 (430485 to 561464) | 3492.2 (3052.2 to 3947.77) | -0.08% (-0.12 to -0.05) |
| Guinea | 129752 (113416 to 146802) | 3105.5 (2720.87 to 3520.65) | 268487 (236777 to 305016) | 3051.02 (2672.43 to 3456.74) | -0.1% (-0.14 to -0.06) |
| Guinea-Bissau | 18630 (16159 to 21054) | 3003.59 (2626.42 to 3397.21) | 38929 (33890 to 44475) | 2933.17 (2573.64 to 3325.83) | -0.1% (-0.13 to -0.07) |
| Guyana | 15729 (13779 to 17810) | 2642.41 (2323.95 to 2985.8) | 19357 (16853 to 21796) | 2624.18 (2295.1 to 2949.78) | -0.03% (-0.03 to -0.02) |
| Haiti | 124618 (108514 to 140250) | 2647.29 (2319.37 to 2986.73) | 283868 (248248 to 324524) | 2629.01 (2304.3 to 2966.16) | -0.05% (-0.06 to -0.03) |
| Honduras | 105632 (91881 to 119823) | 3231.48 (2841.01 to 3645.65) | 288517 (253764 to 326951) | 3261.51 (2882.41 to 3700.26) | 0.04% (0.03 to 0.05) |
| Hungary | 680167 (597203 to 768930) | 5499.6 (4867.26 to 6202.2) | 711005 (623252 to 798738) | 5408.81 (4788.39 to 6085.51) | -0.06% (-0.06 to -0.05) |
| Iceland | 11473 (10088 to 12957) | 4320.83 (3782.84 to 4889.51) | 17532 (15400 to 19673) | 4082.94 (3603.31 to 4611.27) | -0.19% (-0.21 to -0.18) |
| India | 21034411 (18416350 to 23841440) | 3226.78 (2842.31 to 3618.13) | 38580905 (33754640 to 43672640) | 2816.31 (2476.17 to 3169.09) | -0.46% (-0.57 to -0.35) |
| Indonesia | 3825735 (3337840 to 4332317) | 2715.67 (2396.06 to 3054.22) | 7461578 (6488872 to 8462645) | 2637.47 (2322.71 to 2969.76) | -0.05% (-0.07 to -0.03) |
| Iran (Islamic Republic of) | 1731493 (1513550 to 1953080) | 4202.83 (3724.2 to 4725.21) | 3455438 (3053898 to 3953725) | 3879.17 (3430.57 to 4376.33) | -0.21% (-0.25 to -0.17) |
| Iraq | 471347 (410453 to 532663) | 3612.64 (3188.73 to 4069.9) | 1274423 (1111474 to 1448221) | 3570.05 (3138.46 to 4029.41) | -0.04% (-0.05 to -0.03) |
| Ireland | 157502 (138866 to 177698) | 4201.05 (3687.11 to 4753.08) | 250550 (221415 to 281437) | 4140.06 (3683.49 to 4692.32) | -0.09% (-0.11 to -0.07) |
| Israel | 206499 (183040 to 232441) | 4302.53 (3816.42 to 4839.54) | 422118 (370064 to 472101) | 4115.52 (3608.89 to 4634.83) | -0.11% (-0.13 to -0.09) |
| Italy | 2930897 (2588754 to 3276821) | 4162.59 (3684.64 to 4687.58) | 3554242 (3113030 to 3997678) | 4105.26 (3609.92 to 4634.55) | -0.09% (-0.11 to -0.08) |
| Jamaica | 54223 (47294 to 60971) | 2700.91 (2364.63 to 3064.54) | 82258 (72120 to 93002) | 2690.61 (2362.19 to 3041.34) | -0.04% (-0.05 to -0.02) |
| Japan | 7134687 (6286926 to 8008476) | 4808.91 (4269.26 to 5433.48) | 8085995 (7116421 to 9043618) | 4447.66 (3939.3 to 5022.53) | -0.18% (-0.21 to -0.15) |
| Jordan | 94226 (82031 to 107005) | 3633.01 (3182.5 to 4114.72) | 401633 (350042 to 458544) | 3603.73 (3157.81 to 4061.78) | -0.03% (-0.03 to -0.02) |
| Kazakhstan | 584537 (517013 to 657812) | 4007.17 (3553.98 to 4467.35) | 766073 (669185 to 872956) | 4002.02 (3522.07 to 4522.99) | -0.03% (-0.05 to 0) |
| Kenya | 476007 (418171 to 538292) | 3505.44 (3087.9 to 3935.78) | 1242811 (1090711 to 1412001) | 3423.66 (3015.43 to 3854.94) | -0.08% (-0.1 to -0.06) |
| Kiribati | 1496 (1306 to 1707) | 2815.25 (2473.41 to 3188.17) | 2830 (2485 to 3222) | 2870.53 (2520.54 to 3250.4) | 0.12% (0.1 to 0.15) |
| Kuwait | 48509 (41633 to 55381) | 3458.13 (3051.65 to 3874.63) | 175482 (153266 to 204625) | 3585.91 (3169.92 to 4048.18) | 0.12% (0.11 to 0.14) |
| Kyrgyzstan | 142767 (126299 to 161543) | 4023.83 (3539.69 to 4530.48) | 235054 (205762 to 267581) | 3915.41 (3425.12 to 4407.6) | -0.09% (-0.09 to -0.08) |
| Lao People's Democratic Republic | 75073 (65931 to 84184) | 2630.21 (2332.28 to 2948.6) | 157007 (137312 to 178968) | 2496.82 (2196.91 to 2817.64) | -0.18% (-0.19 to -0.16) |
| Latvia | 137689 (121570 to 154752) | 4399.82 (3905.58 to 4956.1) | 116897 (102079 to 131701) | 4361.16 (3838.32 to 4954.22) | -0.03% (-0.04 to -0.03) |
| Lebanon | 92554 (80733 to 103962) | 3552.56 (3116.85 to 3986.96) | 211322 (186215 to 239169) | 3555.98 (3139.45 to 4009.71) | 0.04% (0.03 to 0.06) |
| Lesotho | 31066 (27237 to 35102) | 2911.15 (2564.73 to 3291.88) | 40416 (35061 to 45907) | 2780.57 (2433.54 to 3129.07) | -0.19% (-0.21 to -0.17) |
| Liberia | 48888 (43042 to 55671) | 3020.05 (2646.45 to 3410.93) | 111098 (96708 to 127770) | 2916.17 (2560.41 to 3312.35) | -0.14% (-0.16 to -0.12) |
| Libya | 110554 (96513 to 124867) | 3571.19 (3154.04 to 4009.8) | 250734 (220221 to 286708) | 3579.67 (3165.03 to 4021.26) | -0.02% (-0.03 to -0.01) |
| Lithuania | 184691 (163774 to 208546) | 4482.92 (3990.9 to 5055.34) | 172354 (150606 to 194117) | 4421.96 (3925.15 to 4977.35) | -0.06% (-0.07 to -0.05) |
| Luxembourg | 19243 (17027 to 21783) | 4192.89 (3710.13 to 4750.33) | 33131 (29277 to 37250) | 4045.76 (3581.16 to 4576.69) | -0.11% (-0.12 to -0.1) |
| Madagascar | 259493 (227286 to 294495) | 3402.5 (2980.79 to 3836.91) | 618749 (536049 to 707353) | 3203.19 (2818.27 to 3615.79) | -0.23% (-0.25 to -0.2) |
| Malawi | 198948 (175091 to 223148) | 3282.88 (2906.75 to 3683.75) | 409832 (358653 to 466193) | 3229.74 (2839.4 to 3650.59) | -0.07% (-0.09 to -0.06) |
| Malaysia | 334545 (296587 to 374003) | 2473.78 (2210.8 to 2762.4) | 765979 (670064 to 870864) | 2387.93 (2089.21 to 2695.16) | -0.18% (-0.21 to -0.14) |
| Maldives | 3264 (2842 to 3690) | 2344.07 (2050.58 to 2647.86) | 11492 (9947 to 13404) | 2215.56 (1944.53 to 2490.89) | -0.18% (-0.21 to -0.14) |
| Mali | 152736 (133670 to 171527) | 2728.58 (2404.83 to 3075.63) | 404477 (352978 to 455507) | 2782.99 (2441.41 to 3137.26) | 0.03% (0.01 to 0.05) |
| Malta | 17358 (15309 to 19561) | 4282.52 (3790.09 to 4811.15) | 25751 (22752 to 29091) | 4203.72 (3733.48 to 4757.53) | -0.09% (-0.1 to -0.07) |
| Marshall Islands | 743 (645 to 848) | 2718.83 (2379.38 to 3070.73) | 1288 (1120 to 1462) | 2643.36 (2318.49 to 2985.22) | -0.1% (-0.1 to -0.09) |
| Mauritania | 38448 (33854 to 42920) | 2791.51 (2476.47 to 3126.71) | 85434 (75277 to 96732) | 2769.39 (2439.53 to 3143.33) | -0.08% (-0.1 to -0.05) |
| Mauritius | 24975 (21969 to 28186) | 2625.43 (2329.91 to 2964.99) | 40002 (34794 to 45523) | 2481.51 (2184.37 to 2793.74) | -0.14% (-0.17 to -0.11) |
| Mexico | 1975005 (1720154 to 2245780) | 2987.15 (2631.47 to 3374.96) | 4148822 (3623777 to 4709984) | 3076.45 (2698.66 to 3487.05) | 0.08% (-0.02 to 0.17) |
| Micronesia (Federated States of) | 1963 (1726 to 2210) | 2822.41 (2484.83 to 3181.61) | 2603 (2265 to 2987) | 2826 (2497.14 to 3223.59) | 0.02% (0 to 0.03) |
| Monaco | 1782 (1563 to 2005) | 4098.18 (3635.6 to 4626.35) | 2203 (1917 to 2505) | 3947.85 (3466.44 to 4434.77) | -0.1% (-0.11 to -0.09) |
| Mongolia | 59225 (52202 to 67015) | 3944.64 (3482.69 to 4463.19) | 116204 (101693 to 132992) | 3869.21 (3399.9 to 4350.52) | -0.09% (-0.09 to -0.08) |
| Montenegro | 32680 (28775 to 37087) | 5074.86 (4479.04 to 5735.36) | 39567 (34565 to 44739) | 5093.08 (4514.05 to 5774.75) | 0.02% (0.01 to 0.03) |
| Morocco | 806629 (716966 to 913352) | 3976.88 (3553.33 to 4503.64) | 1478445 (1300908 to 1676720) | 3938.96 (3477.85 to 4452.28) | -0.01% (-0.09 to 0.06) |
| Mozambique | 298390 (261637 to 340092) | 3398.46 (2990.06 to 3842.31) | 646069 (563264 to 733398) | 3357.82 (2953.74 to 3771.47) | -0.03% (-0.04 to -0.03) |
| Myanmar | 693040 (603969 to 782417) | 2247.19 (1973.73 to 2531.46) | 1222920 (1076107 to 1391568) | 2246.83 (1987.45 to 2538.18) | -0.04% (-0.07 to -0.02) |
| Namibia | 26880 (23802 to 30250) | 2919.34 (2581.6 to 3287.04) | 55167 (48879 to 62154) | 2887.36 (2549.17 to 3257.84) | 0% (-0.01 to 0.01) |
| Nauru | 200 (176 to 228) | 2769.73 (2441.52 to 3120.82) | 241 (210 to 274) | 2846.36 (2504.13 to 3189.43) | 0.1% (0.08 to 0.13) |
| Nepal | 602638 (530743 to 687151) | 4309.69 (3817.76 to 4899.51) | 1150825 (1008007 to 1315986) | 4075.32 (3592.08 to 4648.27) | -0.14% (-0.19 to -0.09) |
| Netherlands | 651734 (572785 to 729692) | 3768.02 (3315.4 to 4234.09) | 852227 (747588 to 958469) | 3650.62 (3226.24 to 4086.27) | -0.05% (-0.11 to 0) |
| New Zealand | 193518 (171748 to 217889) | 5277.56 (4666.62 to 5954.51) | 310891 (276478 to 347529) | 4970.24 (4413.58 to 5599.62) | -0.12% (-0.15 to -0.1) |
| Nicaragua | 87463 (76102 to 99466) | 3298.27 (2916.85 to 3729.39) | 202382 (177300 to 231633) | 3256.79 (2861.65 to 3694.18) | -0.01% (-0.02 to 0) |
| Niger | 140905 (122943 to 159740) | 2999.28 (2631.36 to 3391.22) | 429734 (376911 to 487723) | 3033.65 (2667.77 to 3451.21) | 0.07% (0.04 to 0.11) |
| Nigeria | 1889294 (1654211 to 2133205) | 3064.49 (2689.12 to 3457.13) | 4537352 (3952809 to 5139278) | 3008.67 (2638.59 to 3393.45) | -0.06% (-0.1 to -0.02) |
| Niue | 59 (52 to 67) | 2788.36 (2437.53 to 3143.54) | 53 (46 to 60) | 2748.47 (2416.7 to 3098.05) | -0.03% (-0.04 to -0.01) |
| North Macedonia | 97574 (85834 to 110385) | 4836.36 (4259.85 to 5463.47) | 135011 (117412 to 152072) | 4800.67 (4226.44 to 5410.39) | -0.02% (-0.02 to -0.01) |
| Northern Mariana Islands | 1019 (884 to 1186) | 2744.39 (2411.36 to 3105.65) | 1484 (1288 to 1689) | 2722.48 (2393.12 to 3067.97) | -0.04% (-0.06 to -0.01) |
| Norway | 207414 (183343 to 233034) | 4049.05 (3569.57 to 4556.65) | 268732 (237151 to 302101) | 3821.68 (3374.53 to 4301.65) | -0.2% (-0.22 to -0.19) |
| Oman | 49823 (43082 to 57062) | 3481.89 (3051.75 to 3935.56) | 146521 (126649 to 169184) | 3432.48 (3023.46 to 3876.56) | -0.04% (-0.05 to -0.04) |
| Pakistan | 2338401 (2014724 to 2656278) | 3059.35 (2638.02 to 3455.1) | 6112580 (5300163 to 7034456) | 3334.44 (2893.5 to 3769.61) | 0.36% (0.31 to 0.42) |
| Palau | 358 (313 to 406) | 2756.35 (2428.37 to 3113.62) | 606 (528 to 689) | 2690.78 (2377.65 to 3017.67) | -0.06% (-0.08 to -0.04) |
| Palestine | 51045 (44624 to 57749) | 3674.8 (3263.27 to 4161.13) | 148899 (129797 to 168709) | 3585.3 (3161.31 to 4046.14) | -0.07% (-0.08 to -0.06) |
| Panama | 64005 (56103 to 72813) | 3163.82 (2789.12 to 3575.71) | 139114 (121049 to 156834) | 3166.7 (2762.36 to 3573.88) | 0.01% (-0.01 to 0.02) |
| Papua New Guinea | 77360 (67046 to 88080) | 2732.78 (2396.83 to 3106.17) | 214552 (186673 to 245013) | 2706.66 (2373.75 to 3081.35) | -0.02% (-0.04 to 0) |
| Paraguay | 100278 (88123 to 112790) | 3215.79 (2829.75 to 3608.52) | 227547 (198599 to 257032) | 3304.43 (2884 to 3728.56) | 0.08% (0.02 to 0.14) |
| Peru | 417440 (366126 to 474798) | 2481.57 (2174.97 to 2812.31) | 924518 (817651 to 1044053) | 2549.49 (2255.93 to 2872.07) | 0.13% (0.1 to 0.15) |
| Philippines | 1191286 (1043851 to 1345325) | 2653.56 (2335.83 to 2992.76) | 2614041 (2293012 to 2975585) | 2585.87 (2284.09 to 2924.23) | -0.11% (-0.13 to -0.1) |
| Poland | 2234537 (1982193 to 2520771) | 5389.49 (4785.17 to 6052.63) | 2725099 (2392904 to 3060457) | 5274.09 (4671.11 to 5936.96) | -0.09% (-0.1 to -0.08) |
| Portugal | 515061 (455283 to 578100) | 4368.13 (3871.24 to 4935.54) | 642656 (565685 to 722689) | 4258.91 (3767.48 to 4771.06) | -0.11% (-0.13 to -0.09) |
| Puerto Rico | 95521 (84225 to 108118) | 2621.66 (2311.84 to 2971.03) | 120440 (105178 to 135880) | 2614.27 (2304.24 to 2951.9) | -0.03% (-0.04 to -0.02) |
| Qatar | 12648 (10749 to 14877) | 3405.5 (3001.97 to 3851.81) | 101412 (86426 to 119345) | 3467.29 (3051.1 to 3904.59) | 0.01% (-0.01 to 0.03) |
| Republic of Korea | 1752694 (1528643 to 1999909) | 4145.58 (3657.69 to 4686.93) | 2704831 (2366623 to 3045724) | 3871.09 (3409.13 to 4371.71) | -0.18% (-0.21 to -0.16) |
| Republic of Moldova | 201102 (176920 to 227443) | 4457.71 (3931.69 to 5000.98) | 212503 (185638 to 240032) | 4394.68 (3889.95 to 4962.09) | -0.06% (-0.07 to -0.05) |
| Romania | 1381514 (1222241 to 1571008) | 5343.81 (4734.27 to 6064.28) | 1320184 (1149042 to 1490233) | 5150.54 (4540.8 to 5828.94) | -0.15% (-0.17 to -0.14) |
| Russian Federation | 7925263 (7024918 to 8905018) | 4667.59 (4154.67 to 5237.32) | 8715418 (7611600 to 9775837) | 4529.36 (4024.02 to 5083.05) | -0.02% (-0.05 to 0) |
| Rwanda | 154480 (135399 to 176295) | 3481.04 (3047.82 to 3936.36) | 329459 (288626 to 375878) | 3382.48 (2984.53 to 3807.93) | -0.12% (-0.14 to -0.1) |
| Saint Kitts and Nevis | 984 (864 to 1106) | 2651.77 (2308.59 to 2999.54) | 1804 (1554 to 2057) | 2593.21 (2264.34 to 2925.8) | -0.08% (-0.08 to -0.08) |
| Saint Lucia | 3025 (2634 to 3421) | 2761.92 (2408.1 to 3127.45) | 5740 (5020 to 6536) | 2653.96 (2332.07 to 3004.56) | -0.14% (-0.14 to -0.13) |
| Saint Vincent and the Grenadines | 2313 (2015 to 2626) | 2629.4 (2300.37 to 2980.84) | 3364 (2943 to 3808) | 2584.62 (2261.15 to 2919.49) | -0.05% (-0.06 to -0.05) |
| Samoa | 3416 (2967 to 3873) | 2919.3 (2536.92 to 3300.3) | 4847 (4231 to 5492) | 2792.47 (2455.98 to 3144.45) | -0.18% (-0.21 to -0.16) |
| San Marino | 1173 (1033 to 1317) | 4112.63 (3641.15 to 4650.38) | 1825 (1594 to 2061) | 3966.97 (3488.5 to 4454.38) | -0.1% (-0.11 to -0.09) |
| Sao Tome and Principe | 2233 (1953 to 2505) | 2766.92 (2420.98 to 3132.88) | 4479 (3950 to 5096) | 2680.34 (2372.86 to 3030.36) | -0.11% (-0.14 to -0.09) |
| Saudi Arabia | 399358 (348465 to 453519) | 3449.65 (3047.04 to 3890.04) | 1305265 (1122126 to 1526171) | 3508.43 (3089.91 to 3964.15) | 0.07% (0.06 to 0.07) |
| Senegal | 136060 (119606 to 152361) | 2873.89 (2540.8 to 3223.4) | 313133 (274859 to 356947) | 2765.59 (2431.81 to 3136.31) | -0.18% (-0.21 to -0.15) |
| Serbia | 553595 (483422 to 623986) | 5138.33 (4521.81 to 5780.77) | 607813 (533724 to 682177) | 5128.06 (4544.18 to 5764.63) | -0.01% (-0.02 to -0.01) |
| Seychelles | 1552 (1364 to 1750) | 2469.14 (2178.98 to 2794.97) | 2761 (2392 to 3156) | 2334.65 (2050.29 to 2635.09) | -0.18% (-0.19 to -0.17) |
| Sierra Leone | 87875 (76748 to 99378) | 3095.98 (2694.18 to 3499.95) | 179134 (157347 to 202716) | 2930.75 (2563.55 to 3303.54) | -0.19% (-0.22 to -0.16) |
| Singapore | 110595 (97525 to 126162) | 3589.4 (3188.65 to 4035.87) | 244604 (212909 to 278047) | 3329.34 (2896.37 to 3752.56) | -0.12% (-0.18 to -0.07) |
| Slovakia | 295357 (262716 to 335468) | 5210.4 (4652.27 to 5913.21) | 363252 (318123 to 410646) | 5064.94 (4486.86 to 5752.89) | -0.09% (-0.09 to -0.08) |
| Slovenia | 109828 (96955 to 123560) | 4862.21 (4311.94 to 5469.13) | 139292 (121172 to 155746) | 4806.78 (4240.58 to 5425.93) | -0.06% (-0.08 to -0.05) |
| Solomon Islands | 5841 (5085 to 6627) | 2707.23 (2367.71 to 3057.46) | 14709 (12856 to 16832) | 2848.4 (2513.32 to 3234.01) | 0.22% (0.2 to 0.24) |
| Somalia | 149422 (129926 to 170664) | 3249.06 (2857.52 to 3641.81) | 399441 (348364 to 453268) | 3254.04 (2871.68 to 3667.98) | -0.01% (-0.03 to 0) |
| South Africa | 804804 (707604 to 908396) | 2948.76 (2591.41 to 3313.73) | 1491612 (1302877 to 1691659) | 2753.32 (2409.37 to 3110.09) | -0.19% (-0.21 to -0.18) |
| South Sudan | 120669 (105063 to 136741) | 3175.42 (2778.12 to 3574.4) | 199796 (175628 to 226870) | 3150.86 (2777.5 to 3534.73) | -0.04% (-0.05 to -0.02) |
| Spain | 1755641 (1583763 to 1918718) | 3871.75 (3503.26 to 4235.24) | 2247132 (1939446 to 2561406) | 3527.47 (3067.2 to 4007.15) | -0.07% (-0.18 to 0.04) |
| Sri Lanka | 355361 (313634 to 400222) | 2478.71 (2189.89 to 2764.85) | 617959 (537796 to 702447) | 2447.29 (2147.04 to 2782.77) | -0.04% (-0.06 to -0.02) |
| Sudan | 527916 (461415 to 599027) | 3637.42 (3221.26 to 4121.84) | 1234009 (1077083 to 1399951) | 3629.6 (3199.68 to 4073.18) | -0.01% (-0.02 to 0) |
| Suriname | 8623 (7501 to 9724) | 2625.19 (2295.09 to 2961.71) | 16591 (14554 to 18927) | 2663.9 (2346.95 to 3028.23) | 0.06% (0.05 to 0.07) |
| Sweden | 297281 (265443 to 329656) | 2647.8 (2383.99 to 2928.38) | 447647 (388737 to 508289) | 3189.49 (2748.5 to 3610.93) | 0.61% (0.46 to 0.76) |
| Switzerland | 376921 (336909 to 416477) | 4595.39 (4109.18 to 5079.51) | 502393 (438855 to 563448) | 4207.13 (3721.63 to 4728.07) | -0.14% (-0.22 to -0.05) |
| Syrian Arab Republic | 324357 (282729 to 366280) | 3677.31 (3258.52 to 4139.47) | 524527 (457209 to 594343) | 3670.71 (3233.61 to 4127.71) | -0.03% (-0.04 to -0.01) |
| Taiwan (Province of China) | 581029 (525336 to 632456) | 3028.33 (2752.13 to 3287.23) | 1122982 (1023766 to 1231288) | 3328.52 (3064.8 to 3601.76) | 0.37% (0.31 to 0.44) |
| Tajikistan | 141332 (124307 to 159247) | 3832.94 (3382.17 to 4306.88) | 306405 (270417 to 348287) | 3725.9 (3290.62 to 4202.04) | -0.1% (-0.11 to -0.1) |
| Thailand | 1069444 (933052 to 1205036) | 2224.75 (1950.08 to 2489.64) | 2075125 (1796308 to 2348951) | 2284.91 (1998.55 to 2581.55) | 0.14% (0.08 to 0.2) |
| Timor-Leste | 12554 (10972 to 14313) | 2458.4 (2179.58 to 2768.14) | 25279 (21919 to 28444) | 2349.44 (2056.88 to 2653.1) | -0.17% (-0.19 to -0.15) |
| Togo | 67479 (59130 to 77209) | 3117.49 (2749.59 to 3528.76) | 179790 (157054 to 204836) | 2963.73 (2584.15 to 3372.06) | -0.2% (-0.25 to -0.15) |
| Tokelau | 38 (33 to 43) | 2794.71 (2447.23 to 3165.37) | 39 (34 to 44) | 2750.05 (2416.83 to 3111.14) | -0.04% (-0.05 to -0.02) |
| Tonga | 2044 (1796 to 2308) | 2882.44 (2523.31 to 3255.96) | 2518 (2202 to 2824) | 2813.36 (2470.47 to 3164.51) | -0.09% (-0.11 to -0.08) |
| Trinidad and Tobago | 27901 (24472 to 31496) | 2666.52 (2343.9 to 2992.84) | 44414 (38858 to 50338) | 2646.64 (2344.5 to 3001.13) | -0.02% (-0.03 to -0.02) |
| Tunisia | 245008 (215949 to 273068) | 3582.35 (3173.62 to 3997.29) | 472177 (416620 to 536382) | 3647.82 (3228.84 to 4122.66) | 0.05% (0.03 to 0.06) |
| Turkey | 1834059 (1638714 to 2034321) | 3815.23 (3409.68 to 4244.43) | 3441181 (2993295 to 3894469) | 3750.6 (3292.49 to 4240.37) | -0.01% (-0.06 to 0.04) |
| Turkmenistan | 103498 (90493 to 117517) | 3919.63 (3468.78 to 4428.66) | 182615 (160669 to 207483) | 3817.34 (3376.71 to 4295.52) | -0.07% (-0.08 to -0.06) |
| Tuvalu | 224 (195 to 254) | 2846.31 (2497.42 to 3207.47) | 319 (279 to 362) | 2776.87 (2439.6 to 3142.38) | -0.07% (-0.09 to -0.06) |
| Uganda | 344197 (300477 to 384703) | 3363.65 (2973.22 to 3774.87) | 858911 (747682 to 966493) | 3266.14 (2860.24 to 3676.74) | -0.1% (-0.11 to -0.08) |
| Ukraine | 3189021 (2789511 to 3570582) | 5114.95 (4476.43 to 5711.31) | 2982381 (2583005 to 3333472) | 4999.04 (4411.44 to 5580.78) | -0.06% (-0.08 to -0.04) |
| United Arab Emirates | 47357 (40140 to 54898) | 3176.98 (2807.11 to 3536.47) | 341686 (288669 to 406707) | 3252.99 (2867.83 to 3638.56) | 0.04% (0.01 to 0.08) |
| United Kingdom | 2721874 (2413854 to 3060480) | 4035.5 (3581.63 to 4552.43) | 3581700 (3160238 to 4017754) | 4054.15 (3580.24 to 4553.96) | 0.16% (0.11 to 0.21) |
| United Republic of Tanzania | 536448 (469851 to 601762) | 3300.88 (2901.91 to 3703.64) | 1279864 (1127902 to 1449323) | 3204.24 (2821.02 to 3617.48) | -0.08% (-0.09 to -0.07) |
| United States of America | 13405567 (11924745 to 15104332) | 4774.6 (4252.5 to 5367.92) | 18681080 (16946961 to 20396397) | 4501.63 (4092.82 to 4911.62) | -0.05% (-0.11 to 0.01) |
| United States Virgin Islands | 2678 (2361 to 3046) | 2645.26 (2344.73 to 2991.05) | 3190 (2742 to 3632) | 2630.06 (2312.54 to 2981.11) | -0.01% (-0.03 to 0) |
| Uruguay | 133523 (118205 to 149381) | 3966.03 (3504.26 to 4470.33) | 172071 (150937 to 192804) | 4191.52 (3697.03 to 4715.89) | 0.17% (0.12 to 0.21) |
| Uzbekistan | 598472 (524527 to 678144) | 3952.97 (3494.02 to 4460.08) | 1251493 (1092789 to 1427296) | 3910.79 (3446.23 to 4416.18) | -0.01% (-0.02 to 0) |
| Vanuatu | 2920 (2546 to 3313) | 2913.86 (2562.97 to 3293.35) | 7179 (6250 to 8207) | 2945.24 (2584.51 to 3354.19) | 0.03% (0.01 to 0.06) |
| Venezuela (Bolivarian Republic of) | 464834 (406034 to 527830) | 3103.49 (2721.36 to 3498.04) | 869800 (755456 to 976102) | 3014.83 (2623.93 to 3381.14) | -0.11% (-0.12 to -0.1) |
| Viet Nam | 1303478 (1146810 to 1462674) | 2590.65 (2286.95 to 2922.66) | 2682058 (2328042 to 3080451) | 2530.99 (2216.62 to 2879.86) | -0.03% (-0.05 to 0) |
| Yemen | 325650 (284449 to 370197) | 3725.76 (3289.51 to 4223.78) | 923386 (803672 to 1044363) | 3627.32 (3200.54 to 4102.4) | -0.11% (-0.12 to -0.1) |
| Zambia | 132412 (116668 to 147882) | 2832.73 (2504.63 to 3168.58) | 379563 (333590 to 429989) | 3020.48 (2670.15 to 3409.18) | 0.09% (0.04 to 0.14) |
| Zimbabwe | 186411 (164395 to 208943) | 2979.95 (2635.72 to 3356.38) | 333686 (292626 to 377136) | 3104.5 (2717.27 to 3514.34) | 0.16% (0.15 to 0.17) |
