# Supplementary material for: Global burden of low back pain and its attributable risk factors from 1990 to 2021: a comprehensive analysis from the global burden of disease study 2021
Source: Front Public Health. 2024 Nov 13;12:1480779. doi: 10.3389/fpubh.2024.1480779 (PMC11598917; doi:10.3389/fpubh.2024.1480779)
Supplement: Supplementary file 3 [file Table_3.docx]

Table S3. DALYs of Low back pain in 1990 and 2021 for both sexes in 204 countries, with EAPC from 1990 to 2021.

| Location | Num 1990 | ASR 1990 | Num 2021 | ASR 2021 | EAPC CI |
| --- | --- | --- | --- | --- | --- |
| Afghanistan | 71876 (51040 to 96100) | 937.52 (671.26 to 1257.46) | 192329 (134797 to 258340) | 935.42 (667.77 to 1244.03) | 0% (-0.01 to 0.01) |
| Albania | 39825 (28329 to 53824) | 1450.55 (1037.29 to 1949.45) | 50119 (35701 to 67964) | 1465.12 (1042.24 to 1978.49) | 0.04% (0.03 to 0.04) |
| Algeria | 174203 (121908 to 233251) | 943.36 (677.67 to 1275.44) | 394980 (281805 to 531508) | 931.15 (667.12 to 1254.59) | -0.06% (-0.08 to -0.05) |
| American Samoa | 251 (177 to 338) | 735.44 (518.42 to 993.35) | 361 (259 to 486) | 713.99 (511.82 to 955.96) | -0.07% (-0.09 to -0.05) |
| Andorra | 655 (470 to 878) | 1070.06 (766.67 to 1427.3) | 1237 (871 to 1674) | 1034.3 (737.79 to 1388.95) | -0.09% (-0.1 to -0.08) |
| Angola | 55754 (39327 to 74024) | 880.39 (621.7 to 1178.21) | 168042 (118451 to 225847) | 841.77 (608.4 to 1126.17) | -0.16% (-0.19 to -0.12) |
| Antigua and Barbuda | 375 (267 to 501) | 673.71 (481.34 to 907.59) | 700 (501 to 939) | 666.76 (475.19 to 890.54) | -0.03% (-0.04 to -0.01) |
| Argentina | 355105 (252342 to 477353) | 1100.45 (782.75 to 1475.94) | 552287 (391178 to 742471) | 1086.54 (770.8 to 1460.14) | -0.03% (-0.07 to 0) |
| Armenia | 34414 (24373 to 46066) | 1112.25 (792.45 to 1489.87) | 41538 (29817 to 56107) | 1113.27 (803.07 to 1493.35) | 0.05% (0.03 to 0.06) |
| Australia | 252973 (179166 to 338769) | 1370.11 (969.96 to 1833.99) | 404050 (289168 to 547269) | 1255.82 (897.47 to 1688.59) | -0.21% (-0.24 to -0.18) |
| Austria | 93423 (67374 to 124427) | 977.3 (711.48 to 1301.82) | 113411 (80594 to 153070) | 909.12 (654.67 to 1219.52) | -0.06% (-0.12 to 0) |
| Azerbaijan | 61950 (44102 to 82673) | 1030.12 (728.49 to 1370.28) | 118188 (84354 to 160452) | 1041.16 (740.51 to 1404.64) | 0.09% (0.07 to 0.1) |
| Bahamas | 1465 (1041 to 1947) | 677.04 (478.26 to 912.38) | 2893 (2044 to 3917) | 670.25 (472.92 to 904.68) | -0.04% (-0.05 to -0.04) |
| Bahrain | 3719 (2551 to 5000) | 911.63 (644.11 to 1211.56) | 13994 (9818 to 18891) | 888.7 (637.63 to 1185.1) | -0.09% (-0.09 to -0.08) |
| Bangladesh | 765542 (546475 to 1020134) | 1083.42 (779.35 to 1447.71) | 1582987 (1135196 to 2130732) | 1003.98 (722.55 to 1347.79) | -0.18% (-0.25 to -0.11) |
| Barbados | 1823 (1305 to 2450) | 687.58 (493.46 to 926.32) | 2690 (1928 to 3645) | 677.01 (485.88 to 912.16) | -0.04% (-0.05 to -0.04) |
| Belarus | 145232 (103246 to 196117) | 1221.94 (872.13 to 1637.87) | 155738 (112242 to 207330) | 1214.75 (876.3 to 1627.36) | -0.01% (-0.02 to 0) |
| Belgium | 135081 (95948 to 182332) | 1101.37 (784.73 to 1493.45) | 164693 (116610 to 222326) | 1072.35 (768.79 to 1439.85) | -0.08% (-0.09 to -0.07) |
| Belize | 891 (627 to 1187) | 690.24 (486.4 to 933.28) | 2701 (1950 to 3640) | 688.95 (495.01 to 936.64) | 0.02% (0.01 to 0.04) |
| Benin | 23061 (16477 to 30829) | 809.83 (580.48 to 1094.24) | 65291 (45708 to 86804) | 779.45 (545.12 to 1046.31) | -0.13% (-0.17 to -0.1) |
| Bermuda | 462 (332 to 622) | 703.14 (508.48 to 943.89) | 640 (454 to 864) | 692.27 (500.45 to 924.2) | -0.06% (-0.06 to -0.05) |
| Bhutan | 4000 (2825 to 5346) | 987.63 (711.63 to 1324.45) | 7071 (5065 to 9474) | 969.88 (695.08 to 1294.5) | -0.04% (-0.05 to -0.03) |
| Bolivia (Plurinational State of) | 31297 (22283 to 42021) | 681.03 (484.56 to 920.08) | 75178 (53099 to 99696) | 686.44 (486.31 to 916.61) | 0.01% (-0.03 to 0.05) |
| Bosnia and Herzegovina | 61426 (43719 to 82066) | 1333.64 (956.33 to 1782.22) | 62599 (45076 to 83829) | 1372.37 (984.17 to 1838.16) | 0.11% (0.09 to 0.12) |
| Botswana | 5891 (4190 to 7792) | 727.21 (513.26 to 973.37) | 14506 (10259 to 19479) | 714.69 (514.28 to 956.16) | -0.02% (-0.04 to -0.01) |
| Brazil | 1239399 (881976 to 1657077) | 1002.4 (715.13 to 1344.89) | 2539065 (1819608 to 3418673) | 1034.16 (744.86 to 1387.84) | 0.08% (0.05 to 0.1) |
| Brunei Darussalam | 2102 (1465 to 2825) | 1017.13 (730.07 to 1374.63) | 4646 (3301 to 6220) | 972.16 (690.57 to 1302.37) | -0.13% (-0.15 to -0.11) |
| Bulgaria | 150147 (107652 to 202730) | 1415.56 (1020.14 to 1894.13) | 135150 (96976 to 181613) | 1384.98 (989.34 to 1861.48) | -0.04% (-0.05 to -0.04) |
| Burkina Faso | 46458 (32754 to 61933) | 800.79 (570.55 to 1078.02) | 111868 (79167 to 150637) | 785.07 (570.92 to 1068.36) | -0.11% (-0.14 to -0.08) |
| Burundi | 29944 (21345 to 40059) | 890.09 (637.06 to 1201.52) | 69173 (48749 to 92368) | 841.33 (598.81 to 1124.49) | -0.21% (-0.22 to -0.2) |
| C?te d'Ivoire | 59192 (42031 to 78814) | 817.96 (587.68 to 1083.26) | 150384 (106075 to 203961) | 799.33 (570.49 to 1086.16) | -0.06% (-0.11 to -0.02) |
| Cabo Verde | 1874 (1328 to 2498) | 773.46 (542.32 to 1045.71) | 3860 (2732 to 5211) | 721.26 (515.48 to 976.82) | -0.25% (-0.29 to -0.21) |
| Cambodia | 48876 (35114 to 65214) | 746.24 (534.26 to 1001.73) | 105508 (74404 to 140804) | 692.21 (492.69 to 928.92) | -0.25% (-0.27 to -0.24) |
| Cameroon | 54889 (38351 to 73369) | 843.27 (593.96 to 1140.43) | 167339 (118112 to 225928) | 803.38 (571.44 to 1087.33) | -0.17% (-0.21 to -0.14) |
| Canada | 332779 (235195 to 445252) | 1086.6 (769.43 to 1450.19) | 483677 (344637 to 660082) | 990.93 (705.75 to 1328.01) | -0.21% (-0.24 to -0.18) |
| Central African Republic | 14851 (10621 to 19728) | 853.85 (620.59 to 1132.86) | 31005 (22022 to 41247) | 846.45 (605.26 to 1130.73) | -0.04% (-0.05 to -0.03) |
| Chad | 34899 (25016 to 47157) | 930.8 (662.63 to 1258.23) | 83842 (58674 to 110863) | 853.39 (607.2 to 1140.46) | -0.24% (-0.33 to -0.16) |
| Chile | 134995 (96082 to 180167) | 1108.18 (792.09 to 1482.72) | 245014 (173292 to 329867) | 1097.66 (779.01 to 1480.04) | -0.05% (-0.08 to -0.01) |
| China | 7772958 (5520145 to 10545676) | 749.03 (530.01 to 1013.84) | 11297805 (7931468 to 15328056) | 603.03 (427.63 to 810.16) | -0.49% (-0.6 to -0.39) |
| Colombia | 239197 (168594 to 319921) | 909.01 (647.26 to 1215.22) | 493600 (347216 to 661959) | 916.32 (645.9 to 1226.5) | 0.07% (0.04 to 0.09) |
| Comoros | 2269 (1619 to 3026) | 790.53 (567.98 to 1053.96) | 4982 (3591 to 6639) | 797.8 (578.73 to 1064.13) | -0.02% (-0.05 to 0) |
| Congo | 12508 (8847 to 16661) | 807.44 (575.44 to 1074.5) | 33326 (23525 to 44929) | 802.87 (571.11 to 1084.19) | -0.04% (-0.05 to -0.02) |
| Cook Islands | 111 (79 to 149) | 721.99 (513.02 to 975.84) | 160 (113 to 217) | 734.12 (519.82 to 992.78) | 0.1% (0.08 to 0.11) |
| Costa Rica | 21323 (15051 to 28586) | 869.84 (620.43 to 1166.21) | 44770 (32024 to 60052) | 843.89 (603.9 to 1128.09) | -0.08% (-0.09 to -0.08) |
| Croatia | 77873 (55832 to 103325) | 1369.97 (987.74 to 1808.92) | 80639 (57456 to 108284) | 1346.91 (961.41 to 1812.34) | -0.01% (-0.07 to 0.05) |
| Cuba | 75970 (54505 to 102623) | 691.58 (494.03 to 938.56) | 97908 (69968 to 129870) | 645.41 (465.92 to 847.55) | -0.09% (-0.13 to -0.05) |
| Cyprus | 8848 (6240 to 12124) | 1081.42 (761.17 to 1475.52) | 18417 (13136 to 24993) | 1069.96 (768.31 to 1437.91) | -0.06% (-0.08 to -0.04) |
| Czechia | 185222 (131803 to 247476) | 1534.77 (1091.22 to 2045.93) | 219883 (157882 to 295511) | 1488.41 (1068.88 to 2002.2) | -0.09% (-0.1 to -0.09) |
| Democratic People's Republic of Korea | 155361 (109559 to 209162) | 820.1 (578.5 to 1110.88) | 247560 (173494 to 333060) | 771.93 (541.25 to 1033.12) | -0.22% (-0.24 to -0.2) |
| Democratic Republic of the Congo | 204118 (144026 to 274757) | 866.28 (625.15 to 1169.22) | 503220 (359172 to 677111) | 847.33 (608.45 to 1144.54) | -0.09% (-0.11 to -0.06) |
| Denmark | 93137 (65818 to 121968) | 1458.42 (1030.23 to 1916.67) | 100854 (71492 to 137039) | 1276.55 (891.31 to 1730.95) | -0.7% (-0.83 to -0.56) |
| Djibouti | 2019 (1439 to 2721) | 798.55 (571.08 to 1068.1) | 7540 (5330 to 10170) | 753.34 (536.13 to 1015.59) | -0.21% (-0.22 to -0.2) |
| Dominica | 443 (315 to 594) | 708.04 (508.32 to 948.68) | 527 (380 to 706) | 678.83 (491.29 to 903.19) | -0.16% (-0.17 to -0.15) |
| Dominican Republic | 36605 (25924 to 49243) | 673.85 (483.59 to 897.35) | 75554 (53994 to 101485) | 691.71 (495.57 to 930.73) | 0.08% (0.05 to 0.1) |
| Ecuador | 49048 (34823 to 64964) | 651.61 (465.82 to 871.94) | 107611 (76764 to 143498) | 610.22 (434.75 to 815.8) | -0.22% (-0.33 to -0.12) |
| Egypt | 396140 (278915 to 535292) | 943.71 (670.42 to 1267.08) | 866670 (611823 to 1160825) | 964.52 (697.67 to 1290.44) | 0.09% (0.05 to 0.13) |
| El Salvador | 33850 (23847 to 45608) | 844.48 (599.47 to 1140.34) | 54833 (39234 to 73801) | 865.98 (619.19 to 1164.41) | 0.14% (0.12 to 0.15) |
| Equatorial Guinea | 2306 (1651 to 3078) | 853.52 (609.48 to 1140.03) | 7988 (5601 to 10652) | 815.2 (587.33 to 1087.3) | -0.17% (-0.19 to -0.15) |
| Eritrea | 14552 (10372 to 19347) | 745.61 (533.09 to 1001.92) | 34196 (24297 to 45471) | 754.28 (536.42 to 1008.71) | 0.08% (0.06 to 0.09) |
| Estonia | 22244 (15806 to 29980) | 1205.94 (857.45 to 1629.86) | 22599 (16134 to 30164) | 1194.77 (857.26 to 1601.87) | 0.01% (-0.01 to 0.04) |
| Eswatini | 2976 (2135 to 3979) | 659.33 (472.83 to 889.84) | 5284 (3793 to 7049) | 639.2 (456.72 to 853.76) | -0.15% (-0.19 to -0.11) |
| Ethiopia | 265081 (187189 to 353727) | 884.45 (632.09 to 1180.21) | 582836 (412379 to 778457) | 822.39 (583.75 to 1105.62) | -0.22% (-0.24 to -0.21) |
| Fiji | 4042 (2876 to 5362) | 725.03 (516.19 to 973.57) | 6135 (4348 to 8205) | 695.3 (489.51 to 928.93) | -0.12% (-0.13 to -0.12) |
| Finland | 59803 (42338 to 80105) | 981.53 (698.25 to 1313.38) | 73349 (51555 to 99318) | 938.73 (660.92 to 1269.84) | -0.1% (-0.12 to -0.08) |
| France | 724568 (525420 to 973795) | 1071.67 (774.65 to 1445.43) | 951532 (681052 to 1270497) | 1071.05 (771.19 to 1433.02) | -0.02% (-0.04 to 0) |
| Gabon | 5607 (3970 to 7514) | 797.13 (567.01 to 1072.09) | 11395 (8125 to 15264) | 792.72 (566.79 to 1061.33) | -0.01% (-0.01 to 0) |
| Gambia | 4331 (3055 to 5773) | 748.38 (531.64 to 1004.42) | 11353 (8121 to 15140) | 724.18 (519.32 to 967.79) | -0.11% (-0.15 to -0.07) |
| Georgia | 58207 (41396 to 78536) | 974.92 (697.24 to 1311.92) | 45020 (32127 to 60305) | 955.37 (686.64 to 1273.16) | -0.15% (-0.19 to -0.1) |
| Germany | 1323936 (943240 to 1797301) | 1312.59 (935.51 to 1781.99) | 1497385 (1067933 to 2000007) | 1242.99 (887.88 to 1668.43) | -0.11% (-0.13 to -0.08) |
| Ghana | 69642 (49281 to 93213) | 735.56 (522.55 to 984.76) | 175751 (124275 to 234096) | 697.19 (500.4 to 921.37) | -0.2% (-0.23 to -0.17) |
| Greece | 128149 (89895 to 171400) | 1020.65 (724.25 to 1367.05) | 148243 (106019 to 197893) | 1012.27 (734.38 to 1350.32) | -0.06% (-0.09 to -0.03) |
| Greenland | 540 (382 to 727) | 1017.62 (729.35 to 1353.07) | 631 (443 to 850) | 966.25 (692.13 to 1294.26) | -0.1% (-0.13 to -0.07) |
| Grenada | 492 (346 to 654) | 686.77 (489.28 to 917.65) | 770 (544 to 1036) | 674.08 (480.12 to 906.28) | -0.04% (-0.06 to -0.03) |
| Guam | 809 (567 to 1089) | 720.76 (508.91 to 978.14) | 1325 (944 to 1765) | 712.29 (508.36 to 953.84) | -0.02% (-0.03 to -0.01) |
| Guatemala | 54938 (38767 to 72920) | 993.07 (710.9 to 1332.79) | 133095 (93889 to 179558) | 946.74 (667.11 to 1267.22) | -0.09% (-0.13 to -0.04) |
| Guinea | 33029 (23391 to 44535) | 806.82 (576.1 to 1086.46) | 68255 (48445 to 91335) | 798.21 (573.47 to 1061.45) | -0.08% (-0.12 to -0.03) |
| Guinea-Bissau | 4620 (3277 to 6168) | 767.92 (548.54 to 1047.1) | 9748 (6866 to 13099) | 756.12 (535.56 to 1019.5) | -0.08% (-0.11 to -0.04) |
| Guyana | 3934 (2795 to 5188) | 672.87 (483.56 to 896.22) | 4900 (3513 to 6548) | 663.94 (480.87 to 885.87) | -0.04% (-0.04 to -0.04) |
| Haiti | 31028 (21725 to 41783) | 673.74 (479.05 to 911.42) | 71355 (51465 to 95405) | 667.35 (487.35 to 899.05) | -0.05% (-0.07 to -0.02) |
| Honduras | 26982 (18855 to 36464) | 852.64 (603.21 to 1157.56) | 75707 (53970 to 101067) | 863.62 (626.95 to 1158.24) | 0.07% (0.06 to 0.08) |
| Hungary | 198912 (142680 to 267017) | 1593.19 (1145.94 to 2126.47) | 212508 (151934 to 283251) | 1573.52 (1118.61 to 2089.53) | -0.03% (-0.03 to -0.02) |
| Iceland | 3092 (2173 to 4186) | 1161.52 (816.92 to 1568.72) | 4734 (3413 to 6384) | 1089.89 (790.97 to 1466.73) | -0.24% (-0.25 to -0.22) |
| India | 5303845 (3799937 to 7088637) | 824.3 (593.59 to 1106.51) | 9780012 (7003903 to 13127349) | 713.88 (509.42 to 955.04) | -0.48% (-0.6 to -0.36) |
| Indonesia | 994739 (703409 to 1332230) | 715.88 (510.71 to 964.32) | 1985798 (1402418 to 2680164) | 697.12 (496.56 to 936.17) | -0.03% (-0.05 to -0.01) |
| Iran (Islamic Republic of) | 451695 (316798 to 606966) | 1131.51 (810.92 to 1522.57) | 923962 (657330 to 1239276) | 1027.98 (740.46 to 1370.26) | -0.25% (-0.3 to -0.2) |
| Iraq | 119579 (83975 to 159365) | 941.41 (674.42 to 1261.15) | 326494 (231950 to 441521) | 918.4 (660.53 to 1236) | -0.07% (-0.08 to -0.06) |
| Ireland | 41688 (29820 to 55596) | 1112.6 (804.77 to 1483.65) | 67700 (48134 to 91490) | 1105.55 (795.89 to 1485.7) | -0.1% (-0.13 to -0.07) |
| Israel | 54881 (38597 to 74176) | 1148.62 (811.6 to 1553.03) | 113007 (81286 to 151345) | 1099.3 (792.66 to 1477.37) | -0.12% (-0.15 to -0.09) |
| Italy | 775975 (551918 to 1042169) | 1088.79 (783.99 to 1463.08) | 952372 (674066 to 1281968) | 1082.24 (773.35 to 1455.86) | -0.08% (-0.09 to -0.06) |
| Jamaica | 13915 (10030 to 18616) | 704.59 (504.74 to 942.93) | 21509 (15212 to 29077) | 699.91 (494.96 to 947.98) | -0.04% (-0.06 to -0.02) |
| Japan | 1974322 (1399798 to 2673927) | 1313.43 (938.44 to 1765.7) | 2232624 (1590996 to 2995120) | 1208.31 (861.08 to 1627.71) | -0.2% (-0.23 to -0.17) |
| Jordan | 24193 (16995 to 32606) | 957.83 (680.14 to 1277.69) | 104643 (74776 to 141394) | 938.81 (675.77 to 1252.94) | -0.05% (-0.06 to -0.05) |
| Kazakhstan | 153008 (109239 to 203416) | 1061.83 (761.95 to 1407.37) | 202892 (145658 to 274065) | 1061.69 (762.69 to 1425.21) | -0.02% (-0.05 to 0) |
| Kenya | 120635 (85397 to 160365) | 923.01 (659.36 to 1242.2) | 319038 (226680 to 426821) | 898.52 (641.5 to 1209.83) | -0.07% (-0.09 to -0.05) |
| Kiribati | 379 (268 to 509) | 724.11 (519.44 to 976.38) | 730 (516 to 974) | 746.76 (528.15 to 990.85) | 0.18% (0.15 to 0.21) |
| Kuwait | 12624 (8810 to 16988) | 901.49 (642.91 to 1194.89) | 46747 (32727 to 62330) | 933.59 (656.38 to 1248.03) | 0.12% (0.11 to 0.14) |
| Kyrgyzstan | 37288 (26572 to 50175) | 1070.49 (765.13 to 1448.33) | 61447 (44570 to 82294) | 1034.97 (748.9 to 1394.38) | -0.1% (-0.11 to -0.09) |
| Lao People's Democratic Republic | 19072 (13572 to 25297) | 683.44 (490.93 to 913.11) | 40834 (29353 to 54784) | 652.12 (465.49 to 875.89) | -0.16% (-0.18 to -0.14) |
| Latvia | 37779 (26816 to 50234) | 1190.66 (852.1 to 1583.42) | 32434 (23142 to 43017) | 1173 (834.75 to 1566.08) | -0.04% (-0.05 to -0.03) |
| Lebanon | 23889 (16924 to 31812) | 923.72 (657.86 to 1231.36) | 54839 (39417 to 73331) | 917.86 (661.62 to 1228.63) | 0.05% (0.03 to 0.07) |
| Lesotho | 7796 (5601 to 10468) | 746.71 (533.6 to 1001.93) | 9952 (7153 to 13239) | 693.96 (496.87 to 927.91) | -0.29% (-0.31 to -0.26) |
| Liberia | 12015 (8509 to 15946) | 761.82 (541.21 to 1017.7) | 27616 (19600 to 36844) | 740.26 (536.48 to 993.84) | -0.11% (-0.12 to -0.1) |
| Libya | 28269 (20147 to 38014) | 936.89 (672.34 to 1254.35) | 65780 (46473 to 87418) | 927.25 (662.63 to 1239.13) | -0.05% (-0.06 to -0.04) |
| Lithuania | 50757 (36227 to 68287) | 1219.55 (878.72 to 1641.15) | 47889 (34138 to 63555) | 1191.04 (848.63 to 1584.12) | -0.09% (-0.1 to -0.07) |
| Luxembourg | 5145 (3680 to 6899) | 1105.28 (793.62 to 1478.32) | 8945 (6345 to 12128) | 1075.22 (763.67 to 1449.56) | -0.1% (-0.11 to -0.09) |
| Madagascar | 66350 (46860 to 88088) | 899.23 (645.96 to 1208.38) | 157542 (109891 to 213014) | 839.3 (597.59 to 1133.11) | -0.25% (-0.28 to -0.22) |
| Malawi | 49928 (35693 to 66093) | 852.77 (624.46 to 1122.3) | 103907 (72944 to 138488) | 845.6 (598.06 to 1145.23) | -0.03% (-0.05 to -0.02) |
| Malaysia | 84880 (60631 to 111150) | 633.49 (452.96 to 837.88) | 198083 (141269 to 268391) | 615.65 (441.54 to 834.04) | -0.17% (-0.22 to -0.12) |
| Maldives | 809 (575 to 1080) | 594.51 (423.61 to 799.4) | 2954 (2067 to 3978) | 564.98 (396.39 to 750.07) | -0.16% (-0.2 to -0.12) |
| Mali | 37185 (26399 to 49289) | 678.85 (482.01 to 897.41) | 99569 (70866 to 132169) | 705.65 (506.25 to 941.94) | 0.09% (0.07 to 0.12) |
| Malta | 4659 (3318 to 6308) | 1142.63 (818.05 to 1540.66) | 7108 (5049 to 9556) | 1136.64 (823.84 to 1525.48) | -0.09% (-0.12 to -0.06) |
| Marshall Islands | 185 (130 to 245) | 700.97 (494.14 to 939.19) | 326 (228 to 441) | 672.29 (476.86 to 903.54) | -0.14% (-0.14 to -0.13) |
| Mauritania | 9527 (6745 to 12737) | 707.29 (505.11 to 954.17) | 21421 (15225 to 28520) | 710.03 (499.49 to 957.27) | -0.05% (-0.08 to -0.01) |
| Mauritius | 6464 (4614 to 8747) | 684.52 (492.41 to 918.49) | 10431 (7487 to 14145) | 640.48 (457.81 to 865.16) | -0.17% (-0.21 to -0.12) |
| Mexico | 503907 (356499 to 676782) | 777.61 (554.24 to 1048.23) | 1085530 (772664 to 1462111) | 801.94 (572.56 to 1081.9) | 0.08% (-0.03 to 0.19) |
| Micronesia (Federated States of) | 498 (360 to 664) | 737.25 (530.16 to 985.86) | 675 (474 to 907) | 735.97 (521.25 to 983.86) | 0.02% (0 to 0.04) |
| Monaco | 479 (338 to 650) | 1079.06 (772.34 to 1450.87) | 591 (416 to 798) | 1036.84 (741.18 to 1397.53) | -0.12% (-0.12 to -0.11) |
| Mongolia | 15209 (10831 to 20209) | 1044.56 (740.93 to 1401.87) | 30156 (21520 to 40698) | 1012.29 (730.65 to 1363.32) | -0.12% (-0.13 to -0.11) |
| Montenegro | 9114 (6509 to 12374) | 1413.07 (1014.22 to 1906.99) | 11231 (7954 to 15170) | 1422.67 (1010.29 to 1912.61) | 0.06% (0.04 to 0.07) |
| Morocco | 216947 (153332 to 289054) | 1089.72 (787.16 to 1460.3) | 399143 (283978 to 533308) | 1060.29 (756.26 to 1416.19) | -0.05% (-0.15 to 0.06) |
| Mozambique | 75773 (54045 to 100283) | 888.72 (641.16 to 1178.69) | 162939 (115077 to 218833) | 880.34 (634.62 to 1181.94) | 0% (-0.02 to 0.01) |
| Myanmar | 170699 (123112 to 226633) | 560.27 (404.3 to 743.96) | 308676 (217558 to 409158) | 565.48 (396.38 to 750.32) | -0.01% (-0.04 to 0.03) |
| Namibia | 6750 (4806 to 8992) | 752.93 (540.53 to 1006.87) | 14038 (9970 to 18733) | 745.7 (532.92 to 1003.14) | 0.02% (0 to 0.04) |
| Nauru | 51 (36 to 68) | 721.07 (516.51 to 970.86) | 62 (44 to 84) | 740.86 (528.75 to 996.74) | 0.12% (0.09 to 0.14) |
| Nepal | 171798 (120761 to 228514) | 1267.96 (900.2 to 1701.95) | 321833 (227535 to 435105) | 1150.42 (821.7 to 1560.96) | -0.23% (-0.32 to -0.13) |
| Netherlands | 168579 (121731 to 224795) | 966.01 (698.97 to 1282.7) | 221249 (157277 to 296044) | 935.36 (669.04 to 1256.8) | -0.06% (-0.12 to -0.01) |
| New Zealand | 51888 (37039 to 69437) | 1410.49 (1007.95 to 1887.74) | 84337 (60144 to 113925) | 1331.3 (951.02 to 1787.55) | -0.13% (-0.15 to -0.11) |
| Nicaragua | 22365 (15773 to 29798) | 874.61 (624.91 to 1174.44) | 53552 (38189 to 71749) | 864.25 (620.38 to 1156.99) | 0% (-0.01 to 0.02) |
| Niger | 35009 (25129 to 46637) | 770.2 (552.83 to 1033.05) | 108264 (76336 to 144761) | 794.97 (561.03 to 1061.63) | 0.16% (0.13 to 0.2) |
| Nigeria | 475284 (339894 to 635043) | 788.34 (561.27 to 1057.08) | 1145003 (813020 to 1532827) | 779.59 (553.38 to 1051.55) | -0.02% (-0.06 to 0.02) |
| Niue | 15 (11 to 21) | 726.59 (516.95 to 978.09) | 14 (10 to 19) | 710.8 (506.44 to 947.97) | -0.05% (-0.06 to -0.04) |
| North Macedonia | 26436 (18911 to 35576) | 1309.9 (940.46 to 1755.04) | 37104 (26145 to 50264) | 1299.76 (924.62 to 1753.5) | 0% (-0.01 to 0.01) |
| Northern Mariana Islands | 265 (184 to 364) | 719.66 (506.25 to 983.78) | 392 (278 to 533) | 711.05 (506.69 to 962.94) | -0.05% (-0.08 to -0.03) |
| Norway | 54496 (38998 to 73201) | 1055.17 (758.41 to 1420.77) | 70985 (50389 to 95633) | 997.68 (714.29 to 1351.09) | -0.2% (-0.21 to -0.18) |
| Oman | 12689 (8924 to 17126) | 903.19 (643.13 to 1208.98) | 38190 (27011 to 51442) | 885.36 (635.05 to 1175.23) | -0.05% (-0.06 to -0.05) |
| Pakistan | 583257 (410997 to 788716) | 781.04 (553.26 to 1059.82) | 1555725 (1091574 to 2118767) | 861 (604.05 to 1170.82) | 0.44% (0.36 to 0.51) |
| Palau | 92 (65 to 124) | 715.09 (511.12 to 965.56) | 158 (112 to 216) | 692.44 (493.09 to 929.83) | -0.08% (-0.1 to -0.06) |
| Palestine | 13020 (9096 to 17412) | 967.73 (693.19 to 1301.39) | 38117 (26850 to 50891) | 928.5 (662.2 to 1249.64) | -0.12% (-0.13 to -0.11) |
| Panama | 16639 (11723 to 22230) | 833.85 (593.15 to 1120.31) | 36829 (26283 to 49125) | 837.5 (599.55 to 1116.35) | 0.03% (0.01 to 0.04) |
| Papua New Guinea | 19483 (13861 to 26143) | 701.9 (498.24 to 937.1) | 54745 (38935 to 73757) | 697.95 (500.13 to 935.46) | 0.01% (-0.01 to 0.03) |
| Paraguay | 25523 (18243 to 33978) | 835.57 (595.64 to 1111.12) | 59709 (42402 to 79967) | 868.92 (617.82 to 1170.43) | 0.12% (0.05 to 0.19) |
| Peru | 104684 (73842 to 139719) | 634.14 (447.75 to 845.22) | 237038 (167052 to 317742) | 652.64 (458.37 to 876.65) | 0.16% (0.13 to 0.19) |
| Philippines | 306672 (218322 to 411477) | 695.44 (493.09 to 936.64) | 684247 (484100 to 923176) | 678.85 (481.55 to 914.75) | -0.11% (-0.13 to -0.09) |
| Poland | 609194 (436923 to 820031) | 1466 (1048.46 to 1969.5) | 749888 (533070 to 1011310) | 1426.76 (1014.51 to 1919.36) | -0.1% (-0.11 to -0.09) |
| Portugal | 138656 (99000 to 185019) | 1165.15 (835.49 to 1551.67) | 176578 (125999 to 237117) | 1144.57 (814.2 to 1538.15) | -0.12% (-0.15 to -0.08) |
| Puerto Rico | 24601 (17334 to 32870) | 676.15 (476.84 to 903.68) | 31312 (22118 to 42091) | 672.68 (480.9 to 904.16) | -0.03% (-0.05 to -0.02) |
| Qatar | 3303 (2263 to 4472) | 880.9 (626.08 to 1180.55) | 27002 (18950 to 36884) | 895.34 (644.16 to 1193.12) | 0% (-0.01 to 0.02) |
| Republic of Korea | 461880 (326978 to 619749) | 1095.85 (796.8 to 1464.34) | 727108 (516989 to 977658) | 1020.94 (729.04 to 1368.32) | -0.21% (-0.23 to -0.19) |
| Republic of Moldova | 54453 (38892 to 73693) | 1211.99 (861.38 to 1635.92) | 58752 (41950 to 79857) | 1191.87 (855.6 to 1607.42) | -0.09% (-0.11 to -0.07) |
| Romania | 397941 (281793 to 536044) | 1529.05 (1085.41 to 2050.83) | 381364 (271281 to 512846) | 1453.03 (1034.87 to 1959.19) | -0.21% (-0.23 to -0.19) |
| Russian Federation | 2139198 (1531728 to 2879390) | 1254.61 (899.04 to 1690.28) | 2352957 (1685540 to 3172235) | 1206.26 (866.68 to 1623.31) | -0.02% (-0.05 to 0.01) |
| Rwanda | 39816 (27725 to 53196) | 930.51 (665.67 to 1255.89) | 86063 (61046 to 115707) | 904.53 (642.64 to 1219.37) | -0.11% (-0.12 to -0.09) |
| Saint Kitts and Nevis | 249 (175 to 335) | 680.96 (482.25 to 909.63) | 468 (335 to 632) | 663.34 (477.14 to 896.17) | -0.09% (-0.09 to -0.09) |
| Saint Lucia | 771 (546 to 1035) | 718.46 (509.33 to 965.08) | 1497 (1070 to 2007) | 683.54 (493.6 to 915.61) | -0.16% (-0.16 to -0.16) |
| Saint Vincent and the Grenadines | 583 (412 to 791) | 676.93 (481.8 to 916.78) | 867 (620 to 1170) | 661.28 (477.03 to 893.51) | -0.06% (-0.07 to -0.06) |
| Samoa | 881 (625 to 1181) | 772.31 (555.07 to 1037.16) | 1249 (892 to 1669) | 726.72 (518.93 to 970.71) | -0.25% (-0.29 to -0.22) |
| San Marino | 313 (224 to 418) | 1083.81 (776.5 to 1444.22) | 488 (344 to 659) | 1041.65 (745.61 to 1398.2) | -0.12% (-0.13 to -0.11) |
| Sao Tome and Principe | 551 (392 to 735) | 700.6 (499.6 to 946.57) | 1120 (788 to 1510) | 679.24 (475.29 to 916.34) | -0.1% (-0.12 to -0.09) |
| Saudi Arabia | 101266 (71515 to 135749) | 888.35 (629.09 to 1191.43) | 344324 (238493 to 460839) | 906.9 (645.76 to 1204.63) | 0.09% (0.08 to 0.1) |
| Senegal | 33424 (23996 to 44640) | 727.16 (514.52 to 972.49) | 77876 (56287 to 103686) | 701.25 (504.41 to 939.38) | -0.18% (-0.21 to -0.15) |
| Serbia | 156661 (111958 to 210310) | 1442.89 (1043.87 to 1926.01) | 174204 (124316 to 234842) | 1443.06 (1031.71 to 1935.49) | 0.01% (0 to 0.01) |
| Seychelles | 396 (280 to 533) | 636.65 (447.6 to 857.7) | 714 (510 to 960) | 596.72 (430.56 to 798.09) | -0.21% (-0.22 to -0.21) |
| Sierra Leone | 22229 (15853 to 29830) | 800.77 (574.03 to 1082.4) | 45217 (31818 to 60773) | 757.03 (534.97 to 1015.89) | -0.18% (-0.2 to -0.16) |
| Singapore | 28325 (20069 to 37680) | 916.28 (655.97 to 1223.16) | 63350 (44460 to 85507) | 847.08 (603.31 to 1134.9) | -0.13% (-0.18 to -0.07) |
| Slovakia | 84309 (60264 to 113283) | 1484.19 (1062.27 to 1986.01) | 103908 (74393 to 141133) | 1421.02 (1015.78 to 1920.78) | -0.12% (-0.13 to -0.1) |
| Slovenia | 30135 (21418 to 40189) | 1322.39 (942.99 to 1757.63) | 38585 (27465 to 51728) | 1302.04 (925.16 to 1744.64) | -0.06% (-0.08 to -0.04) |
| Solomon Islands | 1471 (1045 to 1973) | 700.97 (499.21 to 952.68) | 3808 (2684 to 5079) | 747.37 (531.64 to 1000.84) | 0.29% (0.26 to 0.32) |
| Somalia | 37263 (26233 to 49535) | 842.71 (602.52 to 1122.08) | 100637 (71402 to 134951) | 854.48 (610.55 to 1153.54) | 0.03% (0.01 to 0.04) |
| South Africa | 203699 (146422 to 272076) | 758.6 (540.03 to 1018.52) | 375306 (268811 to 505397) | 693.42 (497.79 to 927.6) | -0.25% (-0.26 to -0.23) |
| South Sudan | 30078 (21484 to 40046) | 814.22 (583.9 to 1093.01) | 50114 (35405 to 66764) | 810.06 (571.77 to 1078.3) | -0.02% (-0.04 to -0.01) |
| Spain | 422933 (300741 to 556185) | 926.49 (663.12 to 1219.2) | 542892 (383938 to 733436) | 841.91 (597.12 to 1116.99) | -0.06% (-0.18 to 0.05) |
| Sri Lanka | 90233 (64128 to 120903) | 634.71 (452.16 to 850.99) | 159140 (113756 to 214997) | 627.37 (448.55 to 841.75) | -0.04% (-0.06 to -0.02) |
| Sudan | 135212 (94903 to 181113) | 954.81 (679.93 to 1277.93) | 317383 (222150 to 424063) | 948.08 (686.86 to 1259.05) | -0.01% (-0.02 to 0) |
| Suriname | 2194 (1552 to 2952) | 675.69 (477.39 to 911.21) | 4273 (3042 to 5754) | 682.63 (486.16 to 919.22) | 0.06% (0.05 to 0.08) |
| Sweden | 74608 (52973 to 98630) | 660.9 (470.57 to 879.39) | 113838 (80719 to 154383) | 802.64 (571.03 to 1090.96) | 0.62% (0.47 to 0.78) |
| Switzerland | 104615 (76068 to 136848) | 1262.16 (917.81 to 1655.13) | 137762 (97949 to 184244) | 1133.67 (805 to 1518.45) | -0.15% (-0.28 to -0.01) |
| Syrian Arab Republic | 83089 (58812 to 111908) | 972.42 (696.03 to 1315.59) | 137236 (97764 to 185525) | 957.43 (683.15 to 1282.41) | -0.06% (-0.07 to -0.05) |
| Taiwan (Province of China) | 151939 (109677 to 199314) | 794.14 (578.01 to 1036.09) | 322191 (240127 to 419803) | 934.38 (692.61 to 1222.08) | 0.64% (0.54 to 0.73) |
| Tajikistan | 36067 (25431 to 48317) | 1005.75 (710.79 to 1356.67) | 78287 (55786 to 104647) | 966.11 (682.79 to 1306.79) | -0.13% (-0.14 to -0.13) |
| Thailand | 264616 (185676 to 354955) | 553.11 (393.13 to 737.34) | 534708 (377829 to 729711) | 581.13 (413.21 to 789.1) | 0.23% (0.15 to 0.31) |
| Timor-Leste | 3151 (2212 to 4234) | 626.94 (443.82 to 839.33) | 6351 (4516 to 8477) | 598.77 (425.49 to 801.44) | -0.16% (-0.19 to -0.12) |
| Togo | 16916 (11845 to 22722) | 810.52 (571.49 to 1105.72) | 45713 (32252 to 60904) | 768.44 (546.32 to 1029.04) | -0.21% (-0.26 to -0.16) |
| Tokelau | 10 (7 to 13) | 726.28 (512.28 to 983.97) | 10 (7 to 14) | 710.84 (510.49 to 956.07) | -0.04% (-0.05 to -0.03) |
| Tonga | 529 (373 to 716) | 761.47 (544.12 to 1036.83) | 653 (468 to 883) | 735.74 (529.52 to 995.53) | -0.14% (-0.15 to -0.12) |
| Trinidad and Tobago | 7131 (5115 to 9491) | 688.83 (496.66 to 922.22) | 11488 (8290 to 15370) | 678.18 (489.74 to 903.24) | -0.03% (-0.04 to -0.03) |
| Tunisia | 63612 (45294 to 85243) | 945.03 (676.26 to 1267.19) | 124587 (89855 to 167508) | 956.96 (692.22 to 1281.08) | 0.03% (0.02 to 0.05) |
| Turkey | 482117 (347459 to 634220) | 1017.07 (735.78 to 1352.19) | 915900 (651279 to 1235554) | 991.64 (708.1 to 1330.33) | 0% (-0.08 to 0.07) |
| Turkmenistan | 26562 (18992 to 35469) | 1034.36 (748.95 to 1383.46) | 47674 (34011 to 64016) | 1002.04 (715.78 to 1341.61) | -0.08% (-0.09 to -0.07) |
| Tuvalu | 58 (41 to 79) | 743.82 (530.35 to 1003.76) | 83 (60 to 112) | 722.19 (517.92 to 974.61) | -0.05% (-0.07 to -0.03) |
| Uganda | 86617 (61362 to 115681) | 880.33 (625.02 to 1190.84) | 217602 (153887 to 289302) | 860.36 (620.38 to 1149.43) | -0.07% (-0.09 to -0.06) |
| Ukraine | 904082 (648401 to 1219303) | 1431.46 (1018.29 to 1915.15) | 837691 (598526 to 1128216) | 1372.69 (983.05 to 1829.59) | -0.1% (-0.13 to -0.06) |
| United Arab Emirates | 12043 (8269 to 16106) | 802.66 (570.51 to 1067.67) | 89867 (63618 to 122253) | 824.18 (588.58 to 1101.14) | 0.05% (0.01 to 0.09) |
| United Kingdom | 723836 (517492 to 969674) | 1062.56 (762.87 to 1424.91) | 951935 (673695 to 1278999) | 1062.95 (761.91 to 1432.1) | 0.15% (0.09 to 0.2) |
| United Republic of Tanzania | 134298 (95805 to 181888) | 855.43 (616.03 to 1155.26) | 325339 (230445 to 435865) | 836.38 (597.96 to 1130.65) | -0.04% (-0.06 to -0.02) |
| United States of America | 3610495 (2585813 to 4807828) | 1279.74 (919.09 to 1708.34) | 4940706 (3587048 to 6412519) | 1179.08 (856.96 to 1536.41) | -0.1% (-0.16 to -0.03) |
| United States Virgin Islands | 691 (488 to 924) | 683.92 (486.3 to 918.97) | 830 (585 to 1116) | 676.6 (488.41 to 901.07) | -0.03% (-0.04 to -0.01) |
| Uruguay | 34740 (24923 to 46320) | 1029.01 (740.2 to 1370.69) | 45929 (32889 to 61955) | 1106.93 (790.61 to 1483.09) | 0.2% (0.15 to 0.26) |
| Uzbekistan | 154269 (111041 to 204875) | 1046.07 (747.58 to 1401.46) | 327637 (232475 to 443183) | 1028.54 (729.41 to 1385.63) | 0% (-0.02 to 0.02) |
| Vanuatu | 753 (531 to 1009) | 772.1 (550.36 to 1042.12) | 1887 (1338 to 2545) | 784.89 (558.63 to 1052.44) | 0.06% (0.02 to 0.09) |
| Venezuela (Bolivarian Republic of) | 119074 (83406 to 159216) | 808.84 (577.48 to 1094.02) | 226670 (163150 to 306182) | 780.58 (560.54 to 1049.6) | -0.13% (-0.14 to -0.11) |
| Viet Nam | 332523 (238427 to 443286) | 674.96 (488.3 to 903.35) | 713856 (510682 to 964555) | 668.72 (476.11 to 901.6) | 0.04% (0 to 0.07) |
| Yemen | 82183 (57910 to 110803) | 980.24 (701.77 to 1316.88) | 234199 (164680 to 311282) | 940.38 (661.96 to 1251.62) | -0.16% (-0.17 to -0.15) |
| Zambia | 31781 (22745 to 42574) | 702.43 (497.82 to 931.85) | 93908 (67285 to 124668) | 769.13 (554.65 to 1023.92) | 0.16% (0.11 to 0.22) |
| Zimbabwe | 46650 (33235 to 61877) | 772.86 (551.64 to 1030.23) | 85949 (60967 to 114996) | 820.06 (583.4 to 1105.89) | 0.25% (0.24 to 0.27) |
