# Supplementary material for: Global burden of low back pain and its attributable risk factors from 1990 to 2021: a comprehensive analysis from the global burden of disease study 2021
Source: Front Public Health. 2024 Nov 13;12:1480779. doi: 10.3389/fpubh.2024.1480779 (PMC11598917; doi:10.3389/fpubh.2024.1480779)
Supplement: Supplementary file 4 [file Table_4.docx]

***Table 4S Percentage contribution of major risk factors to low back pain ASDALYsR by SDI quintiles and GBD regions, 1990-2021***

| High BMI | 1990 | | | 2021 | | | EAPC, 1990-2021 | | |
| --- | --- | --- | --- | --- | --- | --- | --- | --- | --- |
| Location | Females | Males | Both | Females | Males | Both | Females | Males | Both |
| Global | 8.06% (0.84 to 15.84) | 6.46% (0.7 to 12.58) | 7.49% (0.79 to 14.72) | 12.37% (1.28 to 24.1) | 10.64% (1.11 to 20.74) | 11.74% (1.22 to 22.84) | 28.04% (19.29 to 36.18) | 35.30% (21.76 to 46.05) | 28.67% (19.91 to 35.64) |
| East Asia | 3.41% (0.43 to 6.31) | 3.01% (0.39 to 5.57) | 3.25% (0.41 to 6) | 9.40% (0.98 to 18.54) | 7.62% (0.8 to 14.94) | 8.70% (0.91 to 17.25) | 34.08% (26.76 to 42.2) | 27.33% (15.94 to 34.59) | 30.71% (22.58 to 37.75) |
| Southeast Asia | 3.48% (0.38 to 6.44) | 2.42% (0.29 to 4.56) | 3.13% (0.35 to 5.8) | 7.98% (0.79 to 15.61) | 4.96% (0.51 to 9.3) | 6.98% (0.69 to 13.57) | 53.47% (45.65 to 60.03) | 64.54% (54.26 to 72.65) | 56.64% (48.3 to 62.98) |
| Oceania | 10.40% (1.08 to 20.5) | 7.24% (0.8 to 14.09) | 9.10% (0.95 to 18.07) | 13.08% (1.35 to 25.68) | 10.11% (1.06 to 19.28) | 11.88% (1.23 to 23.24) | 25.71% (16.85 to 36.09) | 39.68% (28.5 to 52.88) | 30.45% (23.19 to 38.98) |
| Central Asia | 12.79% (1.33 to 25.68) | 8.82% (0.91 to 17.44) | 11.47% (1.18 to 22.9) | 16.37% (1.7 to 31.55) | 11.94% (1.24 to 24.13) | 14.76% (1.53 to 28.91) | 129.50% (103.19 to 154.89) | 104.49% (73.76 to 132.89) | 123.14% (97.96 to 142.63) |
| Central Europe | 11.67% (1.19 to 23.22) | 11.18% (1.16 to 22.37) | 11.58% (1.19 to 23.03) | 15.64% (1.6 to 30.35) | 14.23% (1.49 to 28.35) | 15.13% (1.56 to 29.77) | 175.74% (129.51 to 210.49) | 153.06% (111.57 to 186.26) | 167.45% (122.06 to 200.86) |
| Eastern Europe | 13.55% (1.37 to 26.61) | 8.81% (0.88 to 17.21) | 12.18% (1.22 to 23.62) | 18.21% (1.95 to 34.5) | 13.60% (1.43 to 27.28) | 16.79% (1.79 to 32.21) | 34.37% (22.43 to 44.51) | 54.29% (22.3 to 76.96) | 37.83% (21.44 to 47.96) |
| High-income Asia Pacific | 3.76% (0.46 to 7.04) | 3.73% (0.46 to 7.02) | 3.79% (0.47 to 7.08) | 5.44% (0.6 to 10.75) | 6.06% (0.67 to 11.6) | 5.71% (0.63 to 10.93) | 46.00% (37.38 to 53.84) | 38.61% (24.5 to 48.12) | 42.30% (33.58 to 49.32) |
| Australasia | 11.97% (1.18 to 24.31) | 10.33% (1.07 to 20.56) | 11.44% (1.14 to 23.16) | 18.29% (1.88 to 35.64) | 17.10% (1.86 to 33.59) | 17.93% (1.88 to 35.06) | 63.47% (46.72 to 80.21) | 72.89% (45.76 to 89.84) | 67.11% (49.53 to 82.48) |
| Western Europe | 9.45% (0.97 to 18.76) | 8.93% (0.95 to 17.78) | 9.30% (0.97 to 18.33) | 13.80% (1.41 to 27.44) | 12.38% (1.29 to 24.83) | 13.23% (1.36 to 26.76) | 44.64% (24.72 to 65.97) | 62.37% (41.85 to 85.22) | 50.79% (31.94 to 70.61) |
| Southern Latin America | 12.62% (1.28 to 25.11) | 10.43% (1.08 to 21.36) | 11.97% (1.22 to 23.97) | 18.22% (1.92 to 35.2) | 16.45% (1.72 to 32.99) | 17.71% (1.86 to 34.67) | 52.81% (31.81 to 76.4) | 65.60% (40.71 to 85.73) | 56.77% (37.32 to 75.23) |
| High-income North America | 14.95% (1.5 to 29.56) | 13.14% (1.37 to 26.74) | 14.21% (1.45 to 28.8) | 20.16% (2.11 to 37.98) | 19.90% (2.07 to 37.79) | 20.06% (2.09 to 37.97) | 44.37% (32.75 to 56.74) | 57.73% (40.08 to 73.32) | 47.98% (36.22 to 59.44) |
| Caribbean | 10.50% (1.08 to 20.94) | 7.03% (0.73 to 13.76) | 9.20% (0.94 to 18.24) | 15.82% (1.65 to 30.74) | 11.75% (1.2 to 23.18) | 14.32% (1.49 to 27.66) | 34.83% (23.03 to 50.8) | 51.44% (30.42 to 70.55) | 41.16% (26.4 to 56.95) |
| Andean Latin America | 10.25% (1.05 to 20.72) | 7.92% (0.86 to 15.87) | 9.28% (0.97 to 18.6) | 16.75% (1.76 to 32.59) | 13.68% (1.41 to 27.24) | 15.51% (1.62 to 30.5) | 50.62% (39.33 to 62.55) | 67.19% (43.65 to 84.33) | 55.69% (43.02 to 65.64) |
| Central Latin America | 12.46% (1.28 to 25.07) | 9.11% (0.95 to 18.19) | 11.38% (1.18 to 22.92) | 18.10% (1.92 to 34.59) | 15.78% (1.65 to 31.19) | 17.38% (1.83 to 33.46) | 58.19% (43.62 to 72.76) | 113.63% (87.93 to 133.26) | 76.05% (59.61 to 91.1) |
| Tropical Latin America | 10.38% (1.1 to 21.23) | 7.86% (0.83 to 15.79) | 9.47% (0.99 to 19.29) | 15.66% (1.6 to 30.89) | 14.14% (1.47 to 28.03) | 15.14% (1.56 to 29.97) | 89.29% (69.6 to 106) | 109.84% (72.55 to 138.68) | 95.32% (75.02 to 110.7) |
| North Africa and Middle East | 12.62% (1.28 to 24.81) | 7.76% (0.82 to 15.42) | 10.52% (1.08 to 20.6) | 19.97% (2.21 to 36.87) | 16.58% (1.77 to 31.85) | 18.52% (2.02 to 34.71) | 45.30% (33.66 to 56.82) | 73.33% (52.04 to 89.3) | 52.65% (40.09 to 64.59) |
| South Asia | 2.65% (0.31 to 5.01) | 1.74% (0.21 to 3.28) | 2.32% (0.27 to 4.4) | 6.72% (0.66 to 12.91) | 4.56% (0.48 to 8.63) | 6.04% (0.6 to 11.57) | 50.95% (35.7 to 67.65) | 79.91% (60.1 to 101.43) | 59.97% (45.38 to 76.21) |
| Central Sub-Saharan Africa | 3.81% (0.44 to 7.33) | 2.64% (0.31 to 4.88) | 3.34% (0.39 to 6.26) | 8.62% (0.88 to 16.54) | 7.14% (0.68 to 13.48) | 8.00% (0.79 to 15.17) | 64.03% (49.73 to 80.54) | 100.91% (73.46 to 131.22) | 78.70% (62.48 to 96.69) |
| Eastern Sub-Saharan Africa | 4.22% (0.45 to 8) | 2.28% (0.29 to 4.26) | 3.45% (0.39 to 6.56) | 7.98% (0.79 to 15.82) | 4.79% (0.5 to 9.08) | 6.74% (0.68 to 13.16) | 153.90% (111.44 to 189.81) | 162.34% (111.49 to 205.05) | 160.20% (117.35 to 191.34) |
| Southern Sub-Saharan Africa | 15.08% (1.53 to 29.63) | 6.69% (0.71 to 12.93) | 12.10% (1.22 to 23.85) | 21.48% (2.28 to 39.74) | 12.69% (1.24 to 25.17) | 18.25% (1.89 to 34.67) | 126.02% (87.68 to 164.56) | 170.84% (115.19 to 220.87) | 139.63% (102.52 to 176.91) |
| Western Sub-Saharan Africa | 7.02% (0.71 to 13.56) | 3.84% (0.43 to 7.21) | 5.61% (0.59 to 10.65) | 11.51% (1.16 to 22.69) | 7.72% (0.79 to 15.36) | 10.03% (1.01 to 19.93) | 42.39% (32.26 to 55.21) | 89.81% (68.96 to 110.67) | 50.78% (41.38 to 60.99) |
| High-middle SDI | 10.18% (1.05 to 20.02) | 7.43% (0.79 to 14.65) | 9.24% (0.96 to 18.35) | 14.60% (1.53 to 28.26) | 11.68% (1.23 to 23.38) | 13.53% (1.41 to 26.68) | 46.24% (37.23 to 56.43) | 52.60% (40.23 to 62.56) | 48.08% (37.76 to 57.92) |
| High SDI | 10.18% (1.03 to 20.12) | 9.59% (1.01 to 19.2) | 9.99% (1.03 to 19.78) | 14.89% (1.55 to 28.63) | 14.63% (1.53 to 28.19) | 14.80% (1.55 to 28.27) | 101.89% (87.73 to 112.53) | 117.64% (96.5 to 133.52) | 107.43% (91.59 to 118.85) |
| Low-middle SDI | 4.70% (0.51 to 9.05) | 3.06% (0.35 to 5.85) | 4.10% (0.45 to 7.86) | 9.59% (0.99 to 18.52) | 7.41% (0.77 to 14.42) | 8.84% (0.92 to 17.23) | 43.38% (34.29 to 49.53) | 57.25% (38.15 to 67.73) | 46.46% (35.01 to 53.01) |
| Low SDI | 3.86% (0.42 to 7.32) | 2.22% (0.28 to 4.14) | 3.22% (0.36 to 6.1) | 6.82% (0.68 to 13.3) | 5.08% (0.51 to 9.61) | 6.16% (0.62 to 11.91) | 104.02% (87.21 to 118.9) | 141.73% (115.39 to 162) | 115.50% (97.02 to 128.76) |
| Middle SDI | 5.89% (0.64 to 11.58) | 4.34% (0.49 to 8.4) | 5.31% (0.59 to 10.41) | 11.90% (1.23 to 23.18) | 9.45% (0.97 to 18.68) | 11.02% (1.13 to 21.55) | 76.77% (62.94 to 87.82) | 128.69% (92.1 to 153.56) | 91.57% (72.65 to 102.39) |

| Occupational ergonomic factors | 1990 | | | 2021 | | | EAPC, 1990-2021 | | |
| --- | --- | --- | --- | --- | --- | --- | --- | --- | --- |
| Location | Females | Males | Both | Females | Males | Both | Females | Males | Both |
| Oceania | 10.69% (9.23 to 12.44) | 21.89% (19.38 to 24.97) | 15.26% (13.65 to 17.05) | 17.61% (14.49 to 21.91) | 27.26% (23.7 to 31.31) | 21.50% (18.86 to 24.77) | -3.45% (-13.86 to 8.04) | -11.91% (-17.53 to -6.03) | -6.26% (-12.6 to 0.58) |
| East Asia | 35.20% (31.35 to 39.06) | 41.90% (38.35 to 45.52) | 37.54% (34.26 to 40.75) | 28.68% (23.2 to 33.95) | 30.48% (26.56 to 35.2) | 29.29% (25.56 to 33.42) | -13.69% (-21.75 to -3.03) | -13.26% (-18.18 to -7.82) | -12.97% (-17.89 to -7.23) |
| Global | 20.29% (18.74 to 22.02) | 30.85% (28.86 to 32.8) | 24.18% (22.35 to 25.88) | 19.14% (17.52 to 20.78) | 27.06% (25.03 to 28.92) | 22.07% (20.34 to 23.69) | -5.69% (-11.17 to 0.51) | -12.30% (-15.09 to -9.09) | -8.71% (-11.93 to -5.3) |
| Southeast Asia | 26.50% (24.04 to 29.25) | 38.42% (35.72 to 41.37) | 30.29% (27.91 to 32.74) | 25.76% (23.15 to 28.65) | 36.14% (33.23 to 39.08) | 29.05% (26.65 to 31.44) | 64.72% (34.19 to 112.05) | 24.57% (7.33 to 46.9) | 40.90% (22.72 to 62.94) |
| Central Europe | 19.90% (17.96 to 21.97) | 26.38% (24.35 to 28.58) | 22.42% (20.56 to 24.39) | 17.18% (15.18 to 19.19) | 22.89% (20.97 to 24.95) | 19.51% (17.71 to 21.21) | -18.54% (-33.73 to -2.6) | -27.25% (-36.12 to -16.82) | -21.97% (-31.43 to -12.34) |
| Central Asia | 26.30% (23.34 to 29.21) | 35.42% (32.19 to 38.48) | 29.35% (26.72 to 32.12) | 25.39% (22.53 to 28.33) | 31.20% (28.35 to 34.03) | 27.51% (24.89 to 30.05) | -2.80% (-12.21 to 7.35) | -5.92% (-11.29 to 0.12) | -4.10% (-9.78 to 2.26) |
| Eastern Europe | 15.19% (13.24 to 17.41) | 19.92% (17.96 to 22.02) | 16.46% (14.69 to 18.31) | 13.38% (11.66 to 15.24) | 17.98% (16.27 to 19.72) | 14.86% (13.31 to 16.42) | -11.87% (-24.25 to 1.68) | -9.72% (-16.23 to -1.79) | -9.67% (-17.55 to -1.11) |
| Australasia | 14.06% (12.19 to 16.35) | 22.27% (20.23 to 24.44) | 16.77% (15.14 to 18.59) | 13.73% (11.9 to 15.99) | 21.91% (20.01 to 24.26) | 16.38% (14.82 to 18.26) | -1.32% (-13.64 to 13.86) | -9.03% (-17.03 to -0.76) | -4.14% (-11.98 to 4.5) |
| High-income Asia Pacific | 14.60% (13.11 to 16.25) | 22.00% (20.26 to 23.74) | 17.22% (15.68 to 18.68) | 14.41% (12.61 to 16.36) | 20.01% (18.31 to 21.98) | 16.51% (14.97 to 18.03) | 19.34% (12.48 to 27.47) | -7.19% (-10.46 to -3.54) | 3.85% (-0.2 to 7.68) |
| High-income North America | 11.22% (10.21 to 12.37) | 20.15% (18.57 to 21.77) | 15.12% (13.96 to 16.28) | 11.28% (10.22 to 12.49) | 18.30% (17.18 to 19.63) | 14.40% (13.43 to 15.59) | -2.38% (-19.83 to 17.84) | -1.58% (-10.42 to 7.98) | -2.35% (-13.04 to 9.99) |
| Southern Latin America | 8.94% (7.94 to 10.07) | 22.88% (21.28 to 24.61) | 13.41% (12.33 to 14.49) | 12.53% (11.34 to 13.76) | 21.84% (20.24 to 23.59) | 15.45% (14.25 to 16.59) | 55.30% (39 to 73.4) | 21.45% (12.31 to 30.59) | 36.34% (28.11 to 45.86) |
| Western Europe | 9.79% (8.93 to 10.67) | 19.44% (18.05 to 20.77) | 13.81% (12.72 to 14.88) | 11.68% (10.6 to 12.7) | 18.04% (16.5 to 19.48) | 14.34% (13.22 to 15.49) | 40.24% (25.97 to 56.09) | -4.57% (-10.42 to 1.71) | 15.29% (8.11 to 22.88) |
| Caribbean | 9.04% (8.05 to 10.13) | 24.62% (22.53 to 26.74) | 14.93% (13.63 to 16.17) | 13.29% (11.64 to 15.02) | 25.65% (23.32 to 27.63) | 17.90% (16.24 to 19.5) | 0.62% (-9.61 to 10.34) | -9.17% (-15.08 to -2.57) | -4.73% (-10.79 to 1.04) |
| Central Latin America | 11.32% (10.19 to 12.49) | 33.21% (31 to 35.49) | 18.35% (16.98 to 19.62) | 13.53% (12.21 to 14.99) | 29.08% (26.65 to 31.59) | 18.66% (17.2 to 20.17) | 47.03% (30.2 to 68.97) | 4.16% (-2.69 to 11.84) | 19.93% (12.42 to 28.8) |
| Andean Latin America | 13.80% (12.44 to 14.94) | 25.09% (23.15 to 26.97) | 18.44% (17.12 to 19.63) | 21.44% (19.5 to 23.79) | 30.47% (27.91 to 32.93) | 25.14% (23.28 to 27.25) | -11.46% (-15.73 to -6.55) | -14.53% (-17.9 to -11.04) | -12.91% (-15.7 to -9.59) |
| Tropical Latin America | 16.89% (14.13 to 20.57) | 30.78% (26.98 to 34.57) | 22.00% (19.48 to 24.91) | 16.45% (13.65 to 20.02) | 27.85% (24.51 to 31.4) | 20.60% (18.03 to 23.52) | 19.56% (7.79 to 34.94) | -12.43% (-18.03 to -6.12) | 1.69% (-4.69 to 8.45) |
| North Africa and Middle East | 9.06% (7.95 to 10.38) | 27.49% (25.52 to 29.55) | 17.07% (15.64 to 18.58) | 9.49% (8.24 to 10.78) | 23.49% (21.59 to 25.37) | 15.52% (14.08 to 16.84) | -4.89% (-14.26 to 6.69) | -12.27% (-17.4 to -6.67) | -8.68% (-14.6 to -2) |
| South Asia | 20.14% (17.83 to 22.74) | 39.72% (36.52 to 42.91) | 27.04% (24.77 to 29.6) | 17.82% (15.3 to 20.93) | 32.15% (28.86 to 35.51) | 22.32% (20.01 to 24.96) | 4.83% (-7.45 to 18.05) | -14.57% (-19.14 to -9.99) | -9.04% (-14.1 to -4.21) |
| Central Sub-Saharan Africa | 40.26% (34.81 to 45.74) | 39.89% (35.53 to 43.8) | 40.11% (36.21 to 44.28) | 32.25% (27.31 to 37.54) | 32.74% (29 to 36.68) | 32.39% (28.97 to 35.91) | -2.56% (-23.18 to 26.26) | -9.49% (-21.82 to 5.74) | -6.35% (-19.21 to 8.28) |
| Eastern Sub-Saharan Africa | 44.38% (41.09 to 47.66) | 49.98% (46.81 to 53.25) | 46.59% (43.47 to 49.51) | 39.29% (36.69 to 42.01) | 42.72% (39.79 to 45.57) | 40.57% (37.87 to 43.1) | -11.52% (-24.08 to 5.02) | -19.06% (-25.92 to -11.3) | -17.46% (-24.72 to -8.97) |
| Southern Sub-Saharan Africa | 14.93% (13.17 to 16.69) | 23.12% (21.05 to 25.31) | 17.83% (16.22 to 19.42) | 16.71% (15.17 to 18) | 20.28% (18.67 to 21.82) | 18.00% (16.66 to 19.25) | -19.89% (-33.38 to -3.53) | -17.91% (-28.11 to -6.17) | -19.25% (-28.51 to -8.79) |
| Western Sub-Saharan Africa | 34.95% (31.97 to 38.23) | 42.79% (39.67 to 45.9) | 38.25% (35.43 to 40.99) | 33.24% (29.61 to 37.06) | 37.54% (34.44 to 40.54) | 34.93% (31.94 to 37.92) | 11.97% (-0.28 to 25.98) | -12.28% (-18.24 to -5.25) | 0.94% (-5.91 to 8.47) |
| High-middle SDI | 19.60% (17.74 to 21.62) | 28.01% (25.87 to 30.11) | 22.53% (20.6 to 24.46) | 17.30% (15.12 to 19.7) | 23.06% (20.8 to 25.28) | 19.47% (17.55 to 21.48) | -11.74% (-22.71 to -0.77) | -17.67% (-23.22 to -11.64) | -13.58% (-19.47 to -7.32) |
| High SDI | 12.31% (11.29 to 13.36) | 20.62% (19.06 to 21.95) | 15.68% (14.47 to 16.82) | 12.33% (11.36 to 13.34) | 18.98% (17.79 to 20.17) | 15.13% (14.1 to 16.23) | 0.12% (-5.7 to 6.5) | -7.93% (-11.46 to -4.76) | -3.51% (-7.2 to -0.04) |
| Low-middle SDI | 22.17% (20.16 to 24.45) | 40.15% (37.19 to 43.27) | 28.67% (26.42 to 31.02) | 22.27% (20.03 to 24.91) | 34.71% (31.76 to 37.59) | 26.50% (24.23 to 29) | 0.47% (-8.73 to 11) | -13.57% (-18.29 to -8.64) | -7.56% (-12.23 to -1.67) |
| Low SDI | 33.09% (30.54 to 35.53) | 46.81% (43.79 to 49.85) | 38.38% (35.72 to 40.91) | 29.06% (26.67 to 31.34) | 39.74% (36.93 to 42.15) | 33.04% (30.52 to 35.33) | -22.38% (-29.83 to -14.7) | -21.42% (-25.88 to -16.53) | -21.95% (-26.3 to -17.26) |
| Middle SDI | 25.66% (23.39 to 27.91) | 36.68% (34.03 to 39.17) | 29.61% (27.35 to 31.76) | 19.92% (17.94 to 22.05) | 28.82% (26.49 to 31.26) | 23.11% (21.25 to 25.02) | -12.19% (-16.82 to -6.87) | -15.11% (-18.07 to -12.02) | -13.91% (-16.6 to -10.45) |

| Smoking | 1990 | | | 2021 | | | EAPC, 1990-2021 | | |
| --- | --- | --- | --- | --- | --- | --- | --- | --- | --- |
| Location | Females | Males | Both | Females | Males | Both | Females | Males | Both |
| Oceania | 11.13% (6.92 to 15.59) | 21.87% (14.66 to 29.09) | 15.49% (10.07 to 20.98) | 10.64% (6.35 to 15) | 21.30% (14.17 to 28.19) | 14.89% (9.47 to 20.09) | -7.61% (-11.9 to -3.26) | -17.47% (-20.47 to -14.73) | -11.97% (-14.91 to -9.32) |
| East Asia | 5.32% (3.33 to 7.65) | 36.35% (26.21 to 45.16) | 17.39% (12.24 to 22.15) | 3.75% (2.18 to 5.46) | 31.36% (22.05 to 39.58) | 14.96% (10.22 to 19.52) | -31.24% (-33.78 to -28.52) | -21.47% (-23.91 to -19.56) | -24.98% (-27.07 to -23.25) |
| Global | 9.27% (5.98 to 12.64) | 28.15% (19.74 to 35.92) | 16.34% (11.13 to 21.32) | 6.38% (4.04 to 8.81) | 22.11% (15.04 to 28.8) | 12.26% (8.16 to 16.33) | -3.76% (-14.9 to 10.22) | -4.36% (-7.56 to -1.41) | -0.58% (-4.58 to 3.41) |
| Southeast Asia | 4.85% (2.98 to 6.9) | 29.86% (20.85 to 38.23) | 13.03% (8.91 to 17.09) | 3.12% (1.93 to 4.44) | 27.07% (18.76 to 34.79) | 11.03% (7.48 to 14.38) | -35.73% (-43.01 to -26.89) | -9.34% (-12.17 to -6.88) | -15.36% (-18.65 to -12.16) |
| Central Europe | 17.80% (11.75 to 23.83) | 30.60% (21.39 to 39.1) | 22.71% (15.47 to 29.65) | 16.45% (10.76 to 22.12) | 25.26% (17.19 to 32.94) | 20.00% (13.42 to 26.46) | -29.43% (-42.67 to -12.13) | -13.73% (-17 to -10.86) | -13.98% (-18.32 to -9.66) |
| Central Asia | 3.00% (1.83 to 4.37) | 26.53% (18.16 to 34.46) | 11.59% (7.8 to 15.4) | 2.89% (1.72 to 4.22) | 25.38% (17.31 to 33.08) | 11.53% (7.72 to 15.31) | -4.43% (-20.46 to 15.15) | -2.62% (-12.52 to 8.39) | -3.87% (-15.37 to 8.37) |
| Eastern Europe | 6.49% (4.04 to 9.3) | 31.28% (22.27 to 39.52) | 14.19% (9.67 to 18.67) | 9.00% (5.69 to 12.52) | 29.14% (20.41 to 37.29) | 15.62% (10.6 to 20.94) | 38.65% (24.92 to 56.34) | -6.83% (-9.93 to -4.22) | 10.07% (5.44 to 15.38) |
| High-income Asia Pacific | 8.89% (5.44 to 12.64) | 32.16% (22.98 to 40.61) | 17.27% (11.75 to 22.53) | 6.90% (4.2 to 9.8) | 23.12% (15.59 to 30.18) | 13.07% (8.64 to 17.59) | -16.25% (-19.81 to -12.69) | -22.17% (-25.65 to -19.23) | -18.60% (-21.62 to -15.94) |
| Australasia | 17.07% (10.91 to 23.07) | 23.13% (15.4 to 30.43) | 19.09% (12.39 to 25.44) | 13.19% (8.5 to 18.44) | 16.27% (10.56 to 22.22) | 14.23% (9.31 to 19.57) | -27.83% (-37.33 to -17.36) | -22.93% (-27.56 to -18.42) | -25.43% (-31.41 to -19.81) |
| Southern Latin America | 18.70% (12.43 to 24.98) | 23.99% (16.34 to 30.89) | 20.33% (13.71 to 26.83) | 15.82% (10.16 to 21.41) | 20.30% (13.53 to 27.17) | 17.22% (11.15 to 23.1) | -22.71% (-30.62 to -15.51) | -29.65% (-34.87 to -24.44) | -25.45% (-30.94 to -19.96) |
| Western Europe | 18.05% (11.9 to 24.04) | 26.82% (18.44 to 34.67) | 21.47% (14.48 to 28.2) | 15.12% (9.83 to 20.27) | 20.87% (13.83 to 27.45) | 17.48% (11.52 to 23.18) | -15.38% (-23.86 to -5.26) | -15.39% (-21.04 to -10.3) | -15.26% (-21.63 to -8.71) |
| High-income North America | 20.57% (13.52 to 27.5) | 25.18% (17 to 33.1) | 22.53% (15 to 29.94) | 14.75% (9.41 to 20.34) | 18.73% (12.12 to 25.28) | 16.51% (10.65 to 22.55) | -22.32% (-31.54 to -11.46) | -28.12% (-33 to -24.2) | -24.31% (-29.62 to -19.71) |
| Caribbean | 10.59% (6.48 to 14.58) | 20.77% (13.96 to 27.48) | 14.42% (9.35 to 19.42) | 7.64% (4.83 to 10.85) | 16.01% (10.47 to 21.52) | 10.75% (6.93 to 14.77) | -27.92% (-39.66 to -13) | -26.81% (-30.95 to -22.91) | -32.15% (-36.72 to -27.74) |
| Central Latin America | 7.91% (4.99 to 10.99) | 19.10% (12.87 to 25.18) | 11.50% (7.58 to 15.49) | 4.55% (2.86 to 6.43) | 12.40% (8.06 to 16.99) | 7.13% (4.58 to 9.86) | -9.15% (-18.26 to 0.64) | -11.19% (-14.07 to -8.69) | -11.40% (-14.38 to -8.27) |
| Andean Latin America | 3.73% (2.21 to 5.48) | 14.01% (9.07 to 18.94) | 7.94% (5.03 to 10.99) | 3.08% (1.85 to 4.42) | 13.00% (8.3 to 17.65) | 7.12% (4.47 to 9.91) | -28.27% (-33.75 to -22.51) | -25.61% (-29.89 to -21.46) | -26.70% (-30.62 to -22.41) |
| Tropical Latin America | 17.38% (11.2 to 23.31) | 25.55% (17.57 to 33.1) | 20.36% (13.5 to 26.81) | 9.52% (5.8 to 13.48) | 14.31% (8.92 to 19.69) | 11.26% (6.98 to 15.72) | -17.21% (-29.63 to -3.34) | -7.19% (-14.2 to 0.82) | -10.29% (-17.12 to -2.16) |
| North Africa and Middle East | 4.12% (2.54 to 5.89) | 24.75% (17.15 to 32.2) | 13.06% (8.78 to 17.28) | 3.74% (2.25 to 5.34) | 21.98% (14.8 to 29.15) | 11.57% (7.65 to 15.54) | -46.19% (-53.88 to -37.18) | -30.92% (-35.75 to -26.32) | -35.89% (-40.52 to -30.61) |
| South Asia | 4.12% (2.55 to 5.96) | 23.88% (16.38 to 31.08) | 11.12% (7.49 to 14.82) | 2.97% (1.77 to 4.31) | 17.48% (11.66 to 23.1) | 7.54% (4.91 to 10.29) | -42.51% (-48.52 to -35.34) | -35.05% (-38.94 to -30.96) | -38.00% (-42.24 to -33.76) |
| Central Sub-Saharan Africa | 1.63% (0.98 to 2.39) | 13.21% (8.55 to 17.98) | 6.38% (4.11 to 8.81) | 1.49% (0.87 to 2.2) | 11.82% (7.64 to 16.43) | 5.77% (3.7 to 8.05) | -45.20% (-51.1 to -38.37) | -44.00% (-49.53 to -38.9) | -44.71% (-49.54 to -39.6) |
| Eastern Sub-Saharan Africa | 3.06% (1.9 to 4.36) | 14.58% (9.58 to 19.71) | 7.60% (4.96 to 10.43) | 2.55% (1.58 to 3.53) | 11.90% (7.7 to 16.13) | 6.18% (3.99 to 8.4) | -8.45% (-24.4 to 10.27) | -10.56% (-18.25 to -1.9) | -9.53% (-17.9 to -1.21) |
| Southern Sub-Saharan Africa | 9.78% (6.07 to 13.84) | 25.52% (17.21 to 33) | 15.40% (10.18 to 20.67) | 5.26% (3.19 to 7.7) | 17.63% (11.61 to 23.39) | 9.87% (6.41 to 13.56) | -16.47% (-24.33 to -7.95) | -18.41% (-22.19 to -15.05) | -18.72% (-23.1 to -14.7) |
| Western Sub-Saharan Africa | 1.76% (1.07 to 2.58) | 9.98% (6.36 to 13.77) | 5.36% (3.4 to 7.41) | 1.24% (0.74 to 1.82) | 8.85% (5.5 to 12.42) | 4.23% (2.61 to 6.01) | -29.27% (-39.58 to -18.68) | -11.36% (-16.78 to -6.54) | -21.03% (-25.78 to -16.5) |
| High-middle SDI | 9.39% (6.05 to 12.77) | 31.89% (22.61 to 40.11) | 17.61% (12.1 to 22.78) | 8.53% (5.49 to 11.74) | 27.46% (19.15 to 35.37) | 15.81% (10.72 to 20.96) | -24.40% (-32 to -16.31) | -20.60% (-23.87 to -17.96) | -23.04% (-26.32 to -19.58) |
| High SDI | 16.36% (10.69 to 21.94) | 27.46% (19.03 to 35.42) | 20.77% (14.02 to 27.24) | 12.42% (8.01 to 16.87) | 20.69% (13.63 to 27.52) | 15.88% (10.35 to 21.31) | -24.11% (-27.2 to -20.98) | -24.68% (-27.98 to -21.75) | -23.56% (-26.26 to -21.01) |
| Low-middle SDI | 4.44% (2.7 to 6.36) | 23.95% (16.41 to 31.09) | 11.52% (7.72 to 15.24) | 3.31% (1.99 to 4.79) | 19.59% (13.26 to 25.77) | 8.88% (5.85 to 12.07) | -9.19% (-13.96 to -4.03) | -13.88% (-16.39 to -11.93) | -10.21% (-12.93 to -7.98) |
| Low SDI | 4.19% (2.65 to 5.89) | 15.85% (10.54 to 21.19) | 8.69% (5.7 to 11.74) | 3.17% (1.94 to 4.42) | 12.58% (8.13 to 17.06) | 6.69% (4.29 to 9.08) | -25.35% (-33.21 to -15.93) | -18.20% (-21.05 to -15.41) | -22.91% (-26.12 to -19.62) |
| Middle SDI | 5.65% (3.56 to 7.82) | 29.86% (21.12 to 37.74) | 14.55% (10.01 to 18.82) | 3.63% (2.23 to 5.17) | 23.06% (15.96 to 29.87) | 10.59% (7.11 to 14.01) | -35.73% (-40.42 to -30.83) | -22.78% (-25.36 to -20.5) | -27.22% (-29.85 to -24.91) |
